# Novel miR-29b target regulation patterns are revealed in two different cell lines

**DOI:** 10.1038/s41598-019-53868-x

**Published:** 2019-11-25

**Authors:** Wenting Zhao, Lesley Cheng, Camelia Quek, Shayne A. Bellingham, Andrew F. Hill

**Affiliations:** 1 Department of Biochemistry and Genetics, La Trobe Institute for Molecular Science, La Trobe University, Melbourne, VIC 3086 Australia; 2 Department of Biochemistry and Molecular Biology, Bio21 Institute, The University of Melbourne, Melbourne, VIC 3010 Australia

**Keywords:** Gene regulation, RNA metabolism

## Abstract

MicroRNAs (miRNAs) are a class of small non-coding RNAs that regulate gene or protein expression by targeting mRNAs and triggering either translational repression or mRNA degradation. Distinct expression levels of miRNAs, including miR-29b, have been detected in various biological fluids and tissues from a large variety of disease models. However, how miRNAs “react” and function in different cellular environments is still largely unknown. In this study, the regulation patterns of miR-29b between human and mouse cell lines were compared for the first time. CRISPR/Cas9 gene editing was used to stably knockdown miR-29b in human cancer HeLa cells and mouse fibroblast NIH/3T3 cells with minimum off-targets. Genome editing revealed mir-29b-1, other than mir-29b-2, to be the main source of generating mature miR-29b. The editing of miR-29b decreased expression levels of its family members miR-29a/c via changing the tertiary structures of surrounding nucleotides. Comparing transcriptome profiles of human and mouse cell lines, miR-29b displayed common regulation pathways involving distinct downstream targets in macromolecular complex assembly, cell cycle regulation, and Wnt and PI3K-Akt signalling pathways; miR-29b also demonstrated specific functions reflecting cell characteristics, including fibrosis and neuronal regulations in NIH/3T3 cells and tumorigenesis and cellular senescence in HeLa cells.

## Introduction

MicroRNAs are a class of 18–24 nucleotides long small non-coding RNAs that affect cellular gene and protein expression by modulating the stability and translational efficiency of their target messenger RNAs (mRNAs)^1^. miRNAs have been discovered in various organisms, and exhibited their critical roles in diverse pathological processes and as potential therapeutic targets for treating diseases^2,3^. The biogenesis of miRNAs starts with RNA polymerase II or III dependent transcription of miRNA gene in the nucleus, which generates a long primary miRNA (pri-miRNA) that can be several hundred nucleotides to kilobases in length^1,4^. Pri-miRNAs are then cleaved into approximately 70 nucleotides hairpin structured precursor miRNAs (pre-miRNAs), which are transported to cytoplasm and undergo further cleavage to form highly unstable miRNA duplexes^5,6^. The passenger strand of the duplex usually forms miRNA* which usually goes through rapid degradation, whereas the guide strand steers miRNA induced silencing complex (miRISC) to target mRNA transcripts^6^. The nucleotide 2–8 from the 5′ end of mature miRNA is called the seed sequence, which can bind to the 3′ UTR of mRNA through Watson-Crick base pairing and induce translational repression and/or mRNA degradation of target mRNA^1^. Nearly half of miRNA genes are found in tandem within clusters and share the same promoters^1,7^, underpinning the similar regulatory features of these miRNAs.

miR-29b belongs to the miR-29 family, which is consisted of miR-29a, miR-29b-1, miR-29b-2 and miR-29c^8–10^. miR-29b-1 and miR-29b-2 are transcribed from different genome loci but have identical mature sequences, thus they are both termed miR-29b^8–10^. In humans, miR-29a and miR-29b-1 are located at chromosome 7q32, separated by 652 bases, and have the same pri-miRNA transcripts, whereas miR-29b-2 and miR-29c are separated by 507 bases at chromosome 1q32, and are transcribed into the same primary miRNAs^11,12^. They are called miR-29a/b-1 cluster and miR-29b-2/c cluster respectively, due to the close localizations and that they share the same promoters^12^. For mir-29b genes, nucleotide 11–32 forms miR-29b*, and nucleotides 52–74 forms mature functional miR-29b^13^.

miR-29b has been associated with various disorders including fibrotic diseases, cancers, and neurodegenerative diseases^14–16^. miR-29 family members are critical regulators of extracellular matrix (ECM) proteins and signalling pathways associated with fibrosis via targeting collagens, fibrillins, and elastin^16^. miR-29b can support osteoblast differentiation either by inhibiting the accumulation of extracellular matrix proteins COL1A1, COL5A3 and COL4A2, or by directly downregulating inhibitors of osteoblast differentiation, such as HDAC4, TGFβ3, ACVR2A, CTNNBIP1 and DUSP2^17^. Also, miR-29b directly regulates CDK6 (cell cycle dependent kinase 6), which is responsible for retinoblastoma (Rb) protein phosphorylation, in acute myeloid leukemia (AML)^11^, mantel cell lymphoma (MCL)^18^ and in cervical carcinogenesis^19^. In addition, miR-29 family expression is markedly upregulated in normal aging mice and in response to DNA damage, involving a potential miR-29-Ppm1d phosphatase-p53 regulatory feedback loop^20^. miR-29b is highly expressed in brains and has shown dysregulated expression levels in neurodegenerative disorders^21,22^. miR-29b can target BACE1 in sporadic AD patients^21^, in cases of spinocerebellar ataxia 17^23^, in brain development of mice and in primary neuronal cultures^21^. miR-29b was reported to regulated human secreted glycoprotein – progranulin, which is involved in frontotemporal dementia^24^. miR-29b is also among a list of miRNAs that were upregulated in exosomes released from prion disease cell model^25^.

With the function diversity of miR-29b, it is speculated that miR-29b may exhibit cellular environment specific regulation patterns. Studies have shown the cell type/disease specific miRNA signatures^26^. However, there is a lack of studies that examine the specificity of miRNA regulation between two cellular environments. In particular miR-29b, a miRNA that has been implicated in various disease disorders, whereby identifying the regulation patterns of miR-29b would provide reference and guidance for its potential therapeutic usage. In this study we systematically designed and revealed details of the specificity and consistency in miR-29b regulations using the same editing method and experimental approaches. Using this system, we investigated gene regulation via miRNA clusters, mature miRNA generation, and differential gene expression (DEG) profiles induced by miR-29b stable knockdown between two cell lines. This study provides a comprehensive analysis into understanding the regulatory patterns of miR-29b in different cellular environments and species.

## Results

### CRISPR/Cas9 mediated stable knockdown of miR-29b in NIH/3T3 and HeLa cells

Five different human and mouse cell lines were screened for the expression levels of miR-29b (Supplementary 1). The human epithelial cervix adenocarcinoma cells - HeLa cells^27^, and the mouse embryo fibroblast cells - NIH/3T3 cells^28^ were selected to establish miR-29b knockdown clones using CRISPR/Cas9 engineering, due to their robust expression levels of miR-29b and distinct features of these two cell lines (Supplementary 1).

**Figure 1:**
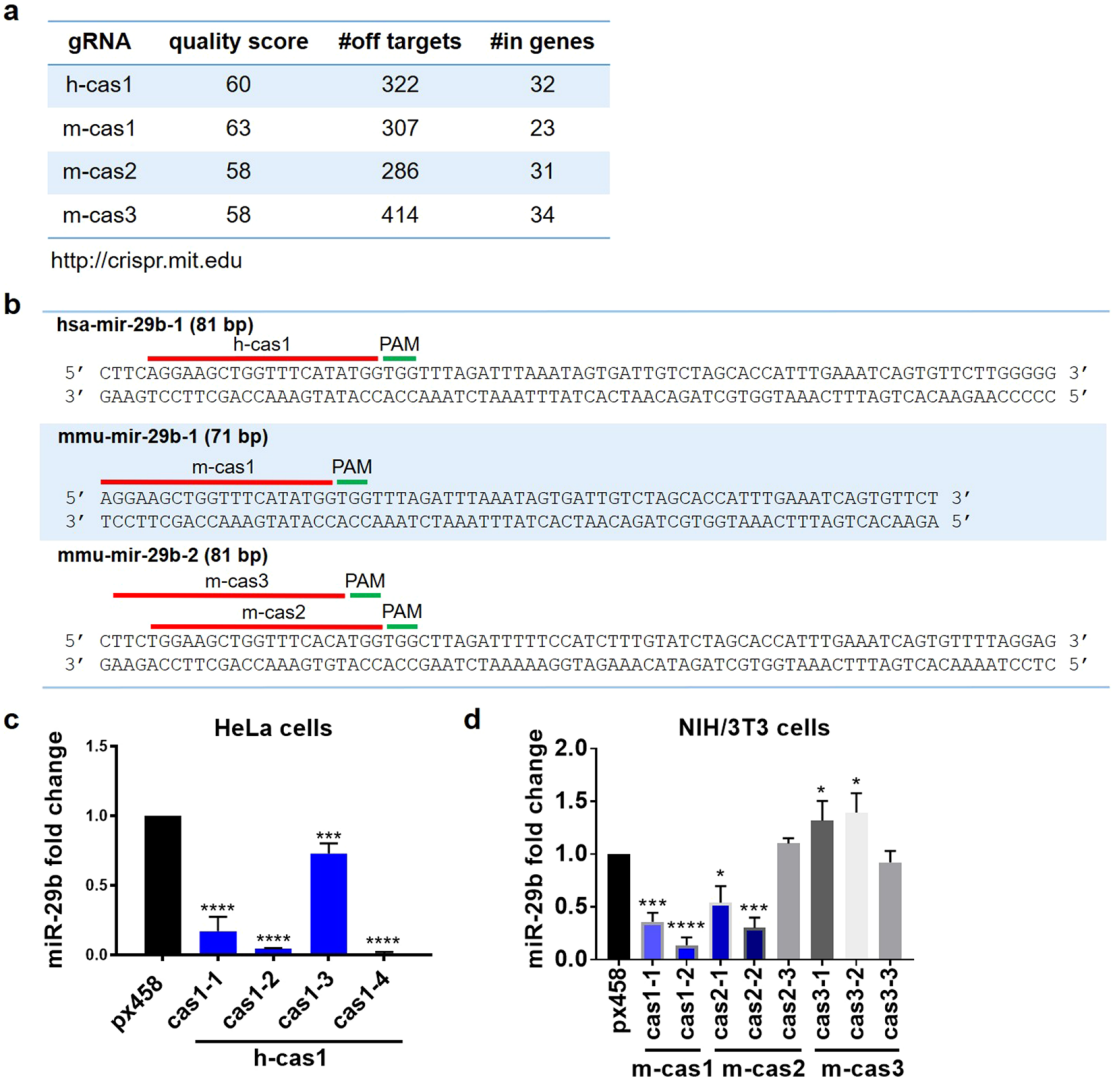
CRISPR/Cas9 mediated miR-29b knockdown in NIH/3T3 and HeLa cells. (**a**) The gRNAs were designed by submitting the whole length of hsa-mir-29b-1, mmu-mir-29b-1, mmu-mir-29b-2 onto gRNA design website http://crispr.mit.edu. The gRNA quality scores were calculated based on the number of potential off-targets and the number of in genes off-targets. (**b**) The gRNAs h-cas1, m-cas1, m-cas2 and m-cas3 locations, sequences and their PAMs were shown on the whole length sequences of hsa-mir-29b-1, mmu-mir-29b-1, mmu-mir-29b-2, respectively. (**c**) The expression levels of miR-29b in HeLa cell clones detected by qRT-PCR. (**d**) The expression levels of miR-29b in NIH/3T3 cells detected by qRT-PCR. Data shown represent the mean values of three independent experiments. Statistical analysis was performed using paired Student’s t-tests. *p < 0.05, ***p < 0.001, ****p < 0.0001.

miR-29b gRNAs were designed by submitting the whole length of miR-29b gene sequences, including hsa-mir-29b-1, hsa-mir-29b-2, mmu-mir-29b-1 and mmu-mir-29b-2, to http://crispr.mit.edu tool. According to gRNA design principles, gRNAs with quality score over 55 have higher specificity and less predicted off-targets when applied for gene editing, and were thus selected for miR-29b editing. The gRNAs, their nucleotide sequences, the targeting localizations on the gene loci, quality scores, numbers of predicted off-targets and ‘in-gene’ off-targets are illustrated (Fig. 1a). Mouse gRNA m-cas1 was designed to target the 5′ end of mmu-mir-29b-1 gene, with 23 potential ‘in-gene’ off-targets out of 307 predicted off-targets; m-cas2 and m-cas3 were used to target mmu-mir-29b-2 at the 5′ end of mmu-mir-29b-2 in NIH/3T3 cells, with 31 and 34 potential ‘in-gene’ off-targets respectively (Fig. 1a,b). H-cas1 has the same nucleotide sequences with m-cas1, and is the only gRNA with quality score over 55 for targeting gene hsa-mir-29b-1, thus is the only gRNA used for target hsa-mir-29b-1 in HeLa cells (Fig. 1a). H-cas1 is predicted to have 322 off-targets, with 32 of them coding for genes (Fig. 1b). No gRNAs with high quality were available for has-mir-29b-2 due to the short length of primary miRNAs.

These four gRNAs were inserted into CRISPR plasmid px458 respectively; the reconstructed plasmids were termed h-cas1, m-cas1, m-cas2 and m-cas3 for further references. The blank vector px458 was used as the cell transfection control. The transfection efficiency of the CRISPR plasmids were tested by monitoring the GFP signal post transfection (Supplementary 2). No difference in miR-29b expression levels were detected following transient transfection in NIH/3T3 cells (Supplementary 2), possibly due to the low transfection efficiency of CRISPR plasmids. FACS was used to isolate cell populations with high and low GFP signals, and qRT-PCR detection showed a significant decrease of miR-29b levels in cell populations with high GFP signals (Supplementary 3). FACS was further used to isolate single cells with high GFP signals into 96-well plates for further culturing them into single cell clones. Once cell clones formed, the culture was expanded and RNA content from these cells were extracted and mature miR-29b expression levels were detected using qRT-PCR.

In NIH/3T3 cells, m-cas1 transfected clones cas1-1 and cas1-2, and m-cas2 transfected clones cas2-1 and cas2-2 exhibited more than 50% decrease of mature miR-29b levels compared to px458 control (Fig. 1d), implying that m-cas1 and m-cas2 were effective at targeting mmu-mir-29b-1 and mmu-mir-29b-2, respectively. M-cas1 transfected clones also exhibited lower levels of miR-29b than m-cas2 clones (Fig. 1d). M-cas3 clones did not show any decrease in mature miR-29b level (Fig. 1d). In HeLa cells, FACS cell sorting was applied following transient transfection of CRISPR plasmids (Supplementary 4). Four cell clones, including cas1-1, cas1-2, cas1-3 and cas1-4, displayed effective knockdown of miR-29b compared to px458 control cells (Fig. 1c); clones cas1-2 and cas1-4 exhibited almost complete knockout of miR-29b (Fig. 1c). These data suggested that gRNAs m-cas1, m-cas2 and h-cas1 facilitated the effective stable knockdown of miR-29b in NIH/3T3 and HeLa cells.

### miR-29b editing is highly specific and reveals mir-29b-1 as the main source of mature miR-29b

#### mir-29b-1, other than mir-29b-2, is the main gene for miR-29b biogenesis

The CRISPR/Cas9 editing of miR-29b in NIH/3T3 cells and HeLa cells was through the non-homologous end joining (NHEJ) mediated repair on the nucleotide sequences of mir-29b-1 and mir-29b-2, resulting in insertions, deletions, or/and mutations of the gene sequences^29^, displayed by the different resultant gene sequences and various expression levels of mature miR-29b in the cell clones.

**Figure 2:**
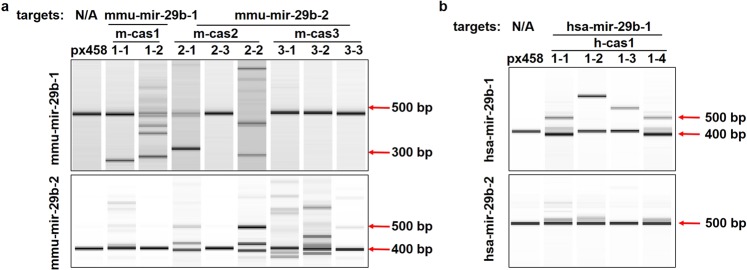
gRNA mediated cleavage reveals mir-29b-1 as the main source of mature miR-29b. (**a**) Surveyor assay detecting the mutations on miR-29b gene locus. mmu-mir-29b-1 was shown to have mutations in cas1-1, cas1-2, cas2-1 and cas2-2; mmu-mir-29b-2 displayed mutations in cas2-1, cas2-2, cas3-1, cas3-2 and cas3-3. (**b**) Surveyor assay showed mutations on hsa-mir-29b-1 and hsa-mir-29b-2 sequences in clone cas1-1, cas1-2, cas1-3 and cas1-4. Surveyor assay products were visualized on Bioanalyser equipment using DNA 1000 chip. Full-length Bioanalyser images for (**a**,**b**) are presented in Supplementary Fig. 5.

Surveyor assay was performed to detect nucleotide changes induced by miR-29b editing^30^. Control group px458 displayed a single band in both cell lines, whereas two or more bands were shown in clones cas1-1, cas1-2, cas2-1 and cas2-2 in NIH/3T3 cells (Fig. 2a). Only single bands were observed in clones cas2-3, cas3-1, cas3-2 and cas3-3 (Fig. 2a), indicating that m-cas1 and m-cas2 effectively targeted mmu-mir-29b-1 gene and induced deletions or insertions on the gene sequences. M-cas3 failed to induce editing on mmu-mir-29b-1 gene and on mature miR-29b production. For mmu-mir-29b-2, clones cas1-1 and cas1-2 displayed single bands with unchanged sizes (Fig. 2a); multiple bands with increased sizes were shown in clones cas2-1 and cas2-2 (Fig. 2a), indicating the insertions on mmu-mir-29b-2 gene. Clones cas3-1, cas3-2 and cas3-3 exhibited multiple bands of mmu-mir-29b-2 gene (Fig. 2a), however, no changes on mature miR-29b levels were observed using qRT-PCR in these clones (Fig. 1c). These data showed that gRNA m-cas1 is effective and specific at downregulating mature miR-29b production, via interrupting mmu-mir-29b-1 sequences; m-cas2 also induced miR-29b downregulation through interrupting both mmu-mir-29b-1 and mmu-mir-29b-2 genes, indicating the non-specificity of gRNA m-cas2; m-cas3 did not induce any cleavage on mmu-mir-29b-1 gene, while insertions or deletions were observed on mmu-mir-29b-2 sequences, suggesting that gRNA m-cas3 is specific at targeting, although no mature miR-29b changes were induced by this gRNA.

In HeLa cells, px458 displayed a single band on hsa-mir-29b-1 gene, while two or more bands were detected in all four HeLa clones (Fig. 2b); bands with increased sizes indicate the nucleotide insertions on hsa-mir-29b-1 nucleotide sequences (Fig. 2b); minor insertions or deletions were also detected as shown in the lower bands of the clones (Fig. 2b). All clones displayed single bands for hsa-mir-29b-2 gene detection (Fig. 2b), suggesting the specificity of gRNA h-cas1 at targeting hsa-mir-29b-1.

In NIH/3T3 cells, gRNA m-cas1 induced stronger decrease of mature miR-29b levels than m-cas2, and in HeLa cells h-cas1 induced almost complete knockout of miR-29b via targeting hsa-mir-29b-1, indicating that mir-29b-1 gene generates more mature miR-29b than mir-29b-2 gene. Indeed, an experiment amplifying the fragment encompassing miR-29a/miR-29b-1 cluster demonstrated that they are co-transcribed as a polycistronic primary transcript^31^. In contrast, miR-29b-2/miR-29c cluster primary transcript or mature miR-29c were not detected, indicating that miR-29b likely derives exclusively from the miR-29a/miR-29b-1 cluster rather than miR-29b-2/miR-29c cluster^31^.

#### miR-29b knockdown caused minimum off-target effect on transcriptome level

Whether miR-29b editing caused off-target effect was also assessed in both cell lines. A list of genes that were predicted to be potential off-targets induced by miR-29b knockdown, based on the mismatches between the genes and the gRNA sequences – with gRNA m-cas1, m-cas2 and h-cas1 predicted off-targets in Tables 1–3 respectively. The scores in Tables 1–3 indicates the chance of the gene to be an off-target. Although the gRNA induced editing and miRNA targeting effect are not driven by the same mechanisms^29,32^, it is worth noting that, the gRNA sequences overlaps with part of the miRNA gene sequences (Fig. 1), thus it is possible that the predicted off-targets can be targeted by miR-29b, its family members miR-29a and miR-29c, or miR-29a*/b*/c*, which are generated from different parts of mir-29 gene sequences^33^.

**Table 1:** Predicted off-targets for gRNAs m-cas1.

gene symbol	UCSC gene	sequence	mismatches	score
Vil1	NM_009509	GGGAAACTGGCTTCCTATGGGGG	4MMs [1:6:11:15]	0.1
Mroh2a	NM_001281466	AGGAAACTGGTGTTATCTGGAGG	4MMs [6:12:14:17]	0
Tfcp2l1	NM_023755	AGGATGGTGGTTTCATAGGATGG	4MMs [5:7:18:20]	0.1
Cfap221	NM_001115074	AAGATGCTGGTTTCACATGGAAG	3MMs [2:5:16]	0.5
Atp2b4	NM_213616	TGGAAGCTGGGGTCATGTGGAGG	4MMs [1:11:12:17]	0.2
mmu-mir-29b-2	NR_029809	TGGAAGCTGGTTTCACATGGTGG	2MMs [1:16]	2.3
Cdkl1	NM_183294	AGGAAGCTGGGGTCATTTGCCAG	4MMs [11:12:17:20]	0.1
Ngly1	NM_021504	AAGATGCTGGTTTTAAATGGGAG	4MMs [2:5:14:16]	0
Cacna1d	NM_028981	AGAAAGCTGGCCTCACATGGTGG	4MMs [3:11:12:16]	0.1
A730017C20Rik	NR_033764	AGAAACCTGGTTTCAGATGCCAG	4MMs [3:6:16:20]	0.1
Rad9a	NM_011237	AGGGAGTTAGTCTCATATGGGAG	4MMs [4:7:9:12]	0.3
Celf2	NM_001160293	AGGAAAATGGTTTAATATTGGAG	4MMs [6:7:14:19]	0
Fkbp1a	NM_008019	AGGCAGCTAGTTTCAGAAGGTGG	4MMs [4:9:16:18]	0
Kcnab2	NM_001252655	AGGAGGCTGGTCTCCTAGGGAGG	4MMs [5:12:15:18]	0
Actr3b	NM_001004365	AGCAAGTTGGTTTCACATGGTGG	3MMs [3:7:16]	0.4
Kdelr2	NM_025841	AGGAGGCTGGCTTCAGCTGGGAG	4MMs [5:11:16:17]	0.1
Vamp1	NM_009496	AGGAAGGTGGTGTCCTATGTTGG	4MMs [7:12:15:20]	0.1
Tmem147	NM_027215	ATGAGGCTGGGTTCAAATGGGGG	4MMs [2:5:11:16]	0.1
Bcl2a1d	NM_007536	AGGAAGATGGCTTCATAAAGAAG	4MMs [7:11:18:19]	0
Bcl2a1a	NM_009742	AGGAAGATGGCTTCATAAAGAAG	4MMs [7:11:18:19]	0
Bcl2a1b	NM_007534	AGGAAGATGGCTTCATAAAGAAG	4MMs [7:11:18:19]	0
Gm1720	NR_046015	AGGAAGCAGGTTTCACATATGGG	4MMs [8:16:19:20]	0

**Table 2:** Predicted off-targets for gRNAs m-cas2.

gene symbol	UCSC gene	sequence	mismatches	score
Cfap221	NM_001115074	AAGATGCTGGTTTCACATGGAAG	3MMs [1:2:5]	2.4
mir-29b-1	NR_029532	AGGAAGCTGGTTTCATATGGTGG	2MMs [1:16]	2.3
Actr3b	NM_001004365	AGCAAGTTGGTTTCACATGGTGG	3MMs [1:3:7]	1.7
Vps53	NM_026664	TGGAAGCTGCCCTCACATGGCAG	3MMs [10:11:12]	0.6
Krtap9-5	NM_001085527	CAGCAGCTGGTTACACATGGTGG	4MMs [1:2:4:13]	0.6
Lrp1b	NM_053011	TGACATTTGGTTTCACATGGAGG	4MMs [3:4:6:7]	0.5
Cacna1d	NM_028981	AGAAAGCTGGCCTCACATGGTGG	4MMs [1:3:11:12]	0.4
Prr14l	NM_194340	TGGACACTGATTTCACATGAAAG	4MMs [5:6:10:20]	0.4
Vmn1r88	NM_001167537	TGGAAGATGGTGTCACTTGGAGG	3MMs [7:12:17]	0.4
Vmn1r86	NM_001167536	TGGAAGATGGTGTCACTTGGAAG	3MMs [7:12:17]	0.4
Slc7a1	NM_007513	CGGAAGCTGATCTCACCTGGTGG	4MMs [1:10:12:17]	0.3
Gm1720	NR_046015	AGGAAGCAGGTTTCACATATGGG	4MMs [1:8:19:20]	0.2
Cd5	NM_007650	GGGCAGGTGGTTTCACAGGGAGG	4MMs [1:4:7:18]	0.2
Zfp786	NM_177882	CGGAAGCTCGTGTCACATGTGGG	4MMs [1:9:12:20]	0.2
Clasp1	NM_177548	GGGAAGCTTGTATCACATTGGGG	4MMs [1:9:12:19]	0.2
Tpi1	NM_009415	TGGAAGGTGATTTCACTTGTCAG	4MMs [7:10:17:20]	0.2
St8sia1	NM_011374	TGGTACCTGGTTTCCCATGTGGG	4MMs [4:6:15:20]	0.1
Il21r	NM_021887	GGGGAGCTGGTTTGACTTGGAGG	4MMs [1:4:14:17]	0.1
Perm1	NM_172417	GGGCAGCTGGTTTCCCAAGGGAG	4MMs [1:4:15:18]	0.1
Efcab7	NM_145549	TGGAAGGTATTTTAACATGGAAG	4MMs [7:9:10:14]	0.1
Gnb3	NM_013530	TGGGAGGTGGTTTCAAATGTGAG	4MMs [4:7:16:20]	0.1
Npr3	NM_001039181	TGGAAGCTGGCTTCCCAGGGAAG	3MMs [11:15:18]	0.1
Tnpo3	NM_177296	GGGAAGTTGGTTTAACTTGGGGG	4MMs [1:7:14:17]	0.1
Mrpl1	NM_001039084	TTGATGCTGGTTTCAAAGGGAAG	4MMs [2:5:16:18]	0.1
Ush2a	NM_021408	TGGAAGCTTGCTTCTCGTGGGGG	4MMs [9:11:15:17]	0
Man2c1	NM_028636	TGGAAGCTGCATTCAGCTGGTGG	4MMs [10:11:16:17]	0
Flna	NM_010227	TGGAAGGTGGTGTCACAGGTAGG	4MMs [7:12:18:20]	0
Otop1	NM_172709	CGGAAGCTGGATTTACAGGGTGG	4MMs [1:11:14:18]	0
Atp2b4	NM_213616	TGGAAGCTGGGGTCATGTGGAGG	4MMs [11:12:16:17]	0
Lrrc2	NM_028838	TGGAACCTGGTTTCCCACTGTGG	4MMs [6:15:18:19]	0

**Table 3:** Predicted off-targets for gRNAs h-cas1.

gene symbol	UCSC gene	sequence	mismatches	score
has-mir-29b-2	NR_029518	TGGAAGCTGGTTTCACATGGTGG	2MMs [1:16]	2.3
EFR3A	NM_015137	AGAAAGCTTTCTTCATATGGTGG	4MMs [3:9:10:11]	0.4
GTF2IRD1	NM_001199207	AGGGGGCTTGTCTCATATGGTGG	4MMs [4:5:9:12]	0.4
ALKBH3	NM_139178	ATGAATCTGCTTTCATGTGGAGG	4MMs [2:6:10:17]	0.3
SCFD1	NR_047547	AAGGAGCTTGTTTCATATCGTGG	4MMs [2:4:9:19]	0.3
DTL	NM_016448	ATGAAGCTGCCTACATATGGAAG	4MMs [2:10:11:13]	0.3
TOR1AIP2	NM_145034	AGGAAACTGCCCTCATATGGCAG	4MMs [6:10:11:12]	0.2
PACS1	NM_018026	AGGAAGATGCTGTCATATGCGAG	4MMs [7:10:12:20]	0.2
LOC100130691	NR_026966	AGGGAGCAGGGTTCATAAGGAGG	4MMs [4:8:11:18]	0.2
TROAP	NM_001278324	AGGCTGCTGGGTTCATAGGGAGG	4MMs [4:5:11:18]	0.2
CPSF4L	NM_001129885	TGAAAGCTGGCTTCACATGGAGG	4MMs [1:3:11:16]	0.1
WDR59	NM_030581	AGGCATCAGGTTTTATATGGAAG	4MMs [4:6:8:14]	0.1
SORL1	NM_003105	AACAAGCTGGTCTCAGATGGTGG	4MMs [2:3:12:16]	0.1
ACTA2	NM_001141945	TGGAGGCTGGCTTGATATGGAAG	4MMs [1:5:11:14]	0.1
KRT76	NM_015848	AGGAAGCTGACTTCATCTGTCAG	4MMs [10:11:17:20]	0.1
H3F3B	NM_005324	AGGAAACTGGTTTCATACTGAAG	3MMs [6:18:19]	0.1
DLGAP4	NM_001042486	TGGCAGCTGGTTTCAGCTGGTGG	4MMs [1:4:16:17]	0.1
TFCP2L1	NM_014553	AGGATGGTGGTTTCATAGGACGG	4MMs [5:7:18:20]	0.1
TAF2	NM_003184	AGCAAGCTGATTTTATGTGGCAG	4MMs [3:10:14:17]	0.1
PPM1F	NM_014634	TGGAAGCTGCTTTAATATTGGGG	4MMs [1:10:14:19]	0.1
ABCB11	NM_003742	AGGAAGCTGGTTTCACTTGAAAG	3MMs [16:17:20]	0.1
GPR142	NM_181790	AGGCAGCTGGATTCACCTGGCGG	4MMs [4:11:16:17]	0.1
WFDC8	NM_181510	AGGAAGCTGTCTCCATAGGGAAG	4MMs [10:11:13:18]	0.1
MLLT6	NM_005937	AGGAAGCTGTTCTTATCTGGGGG	4MMs [10:12:14:17]	0
CELF2	NM_001025076	AGGAAAATGGTTTAATATTGGAG	4MMs [6:7:14:19]	0
RPS6KA5	NM_004755	AGGAAGCTTGTTTCAGCTGTAAG	4MMs [9:16:17:20]	0
SELE	NM_000450	AGGAAGCTGCCTTCAAAGGGTAG	4MMs [10:11:16:18]	0
TET2	NM_001127208	AGGCAGCTGGTTTGCTGTGGTGG	4MMs [4:14:15:17]	0
CNTNAP3	NM_033655	AGGAACCTGGCTTCAAAGGGAGG	4MMs [6:11:16:18]	0
CNTNAP3B	NM_001201380	AGGAACCTGGCTTCAAAGGGAGG	4MMs [6:11:16:18]	0
JAK1	NM_002227	AGAAAGCTGGTTCTACATGGGGG	4MMs [3:13:14:16]	0

Whole transcriptome RNA sequencing is a standard method to examine the off-target effect on transcriptome level caused by CRISPR/Cas9 editing^34^. It was performed in NIH/3T3 clones cas1-1, cas1-2, cas2-1 and cas2-2, and HeLa clones cas1-1, cas1-2, cas1-3 and cas1-4, with px458 as controls in both cell lines. Differential gene expression (DGE) assays were performed on genes with over 20 transcripts reads, with expression data of each clones were compared to px458 control groups in each cell line, respectively; DGE data with p value less than 0.05 were used for further analysis.

In the off-target gene list of NIH/3T3 clones, mmu-mir-29b-2 gene was one of the off-targets for m-cas1 gRNA, with two nucleotide mismatches to the sequence of gRNA m-cas1 (Table 1); mmu-mir-29b-1 gene has two mismatches to m-cas2 gRNA sequences at sites 1 and 16 (Table 2). Due to the sequence similarities between the m-cas1 and m-cas2, some of the off-targets were common to both gRNAs (Tables 1 and 2). Among the predicted off-target genes, Vil1, Cdkl1, Celf2, Vamp1 and Otop1 were predicted to be targeted by miR-29a* in their 3′ UTRs (Tables 1 and 2); Cacna1d, Zfp786, Il21r, and Npr3 can potentially be targeted by miR-29b*, while Clasp1, St8sia1 and Tnpo3 are likely to be targeted by both miR-29a* and miR-29b*; miR-29c* has predicted binding sites in the 3′ UTR of Mrpl1 and Lrrc2 (Tables 1 and 2). This is likely due to that the gRNAs were designed to target the 5′ end of mmu-mir-29b-1 and mmu-mir-29b-2 genes (Fig. 1a), which partially overlap with the seed sequences of miR-29a*/b*/c*, and therefore share common mRNA targets.

**Figure 3:**
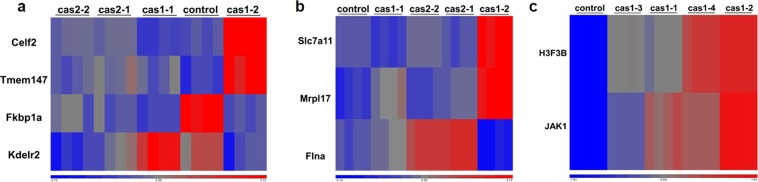
Expression levels of predicted off-targets in NIH/3T3 and HeLa cell clones. Gene differential expression assay was performed on genes with over 20 reads, p value was less than 0.05. (**a**) Among the off-targets predicted for m-cas1, only Celf2, Teme147, Fkbp1a and Kdelr2 were significantly different among the clones. Celf2 and Teme147 were increased significantly in cas1-2 compared to control, while no difference was observed among other clones; Fkbp1a was downregulated in all clones, while Kdelr2 showed down or upregulation in clones compared to control. (**b**) Among the off-targets predicted for m-cas2, Slc7a11, Mrpl17 and Flna were differentially expressed. Slc7a11 and Mrpl17 were significant upregulated in cas1-2, while no changes detected in other clones. Flna was upregulated in cas2-1 and cas2-2, and no changes were found in other clones. (**c**) Among the off-targets predicted for h-cas1, H3F3B and JAK1 were upregulated in HeLa cell clones.

From the RNA sequencing data analysis, four genes in the off-target list for m-cas1, including Celf2, Tmem147, Fkbp1a and Kdelr2 were found to have over 20 transcripts reads for DEG analysis (Fig. 3a). Celf2 and Tmem147 both have 4 mismatches compared with the m-cas1 gRNA sequence and were shown to increase significantly in clone cas1-2, while no changes were detected in other clones compared to px458 (Fig. 3a). Kdelr2 was slightly upregulated in clone cas1-1 compared to px458 (Fig. 3a); Fkbp1a was significantly downregulated in all the clones (Fig. 3a), and it was predicted to be targeted by miR-29a/b/c (Table 1), implying a positive correlation between miR-29b and Fkbp1a. For the m-cas2 off-targets, Slc7a11 and Mrpl1 were upregulated in clone cas1-2, while no changes were detected in other clones compared to px458 (Fig. 3b); these two genes were also predicted to be targeted by miR-29b (Table 2). Flna, with 4 mismatches with m-cas2 at sites 7, 12, 18, and 20 respectively, was increased in clones cas2-1 and cas2-2 compared to the px458 control (Fig. 3b). These data demonstrated that a large part of the predicted gRNA off-targets have potential binding sites for miR-29a*/b*/c* in their 3′ UTRs, which share sequence similarities with the gRNAs themselves; most of these genes were expressed at such low levels that cannot be detected; a few genes including Celf2, Tmem147, Scl7a11 and Mrpl17 exhibited clone cas1–2 specific expression level changes (Fig. 3a,b), probably due to the large miR-29b knockdown extent in clone cas1–2 compared to other clones. Fkbp1a is the only gene that was downregulated in all the NIH/3T3 clones compared to px458 (Fig. 3a); it was also predicted to be targeted by miR-29b (Table 1), implying that Fkbp1a is a potential miR-29b target, instead of the ‘off-target’ in NIH/3T3 cells.

In the HeLa cell clones, hsa-mir-29b-2 gene is shown to have two mismatches on its sequences compared to gRNA h-cas1 (Table 3), therefore it has a high risk of being affected by h-cas1 mediated miR-29b editing. Multiple genes, including EFR3A, GTF2IRD1, ALKBH3, DTL, TOR1AIP2, TROAP, WDR59, SORL1, H3F3B, DLGAP4, TFCP2L1, TAF2, PPM1F, MLLT6, CELF2, SELE, RPS6KA5, TET2, CNTNAP3, CNTNAP3B and JAK1, were predicted to be targeted by miR-29a*, b*, c*, a, b, and/or c in the 3′ UTRs (Table 3). From RNA sequencing data analysis, H3F3B and JAK1 were the only two ‘off-targets’ that were dysregulated in all four HeLa clones compared to px458 (Fig. 3c). The nucleotide sequences of H3F3B are shown to have 3 mismatches compared to the sequences of h-cas1, at sites 8, 18 and 19 respectively; JAK1 has 4 mismatches at sites 3, 13, 14 and 16 respectively (Table 3); H3F3B and JAK1 were predicted to be targeted by miR-29a* and miR-29a*/b*, respectively (Table 3). * miRNAs usually have low expression levels, whereas some * miRNAs have been reported to be functional^35^. It is speculated that upregulated H3F3B and JAK1 were potentially caused by either miR-29a*/b* targeting, or the binding of miR-29b in the mRNA coding region/5′ UTR, or via intermediate regulators. These data indicated that most of the predicted off-targets were not affected by miR-29b editing, suggesting the minimum off-target effect and highly specific editing.

### miR-29b editing potentially decreases miR-29a/c levels by disturbing the tertiary structures of miRNA clusters

**Figure 4:**
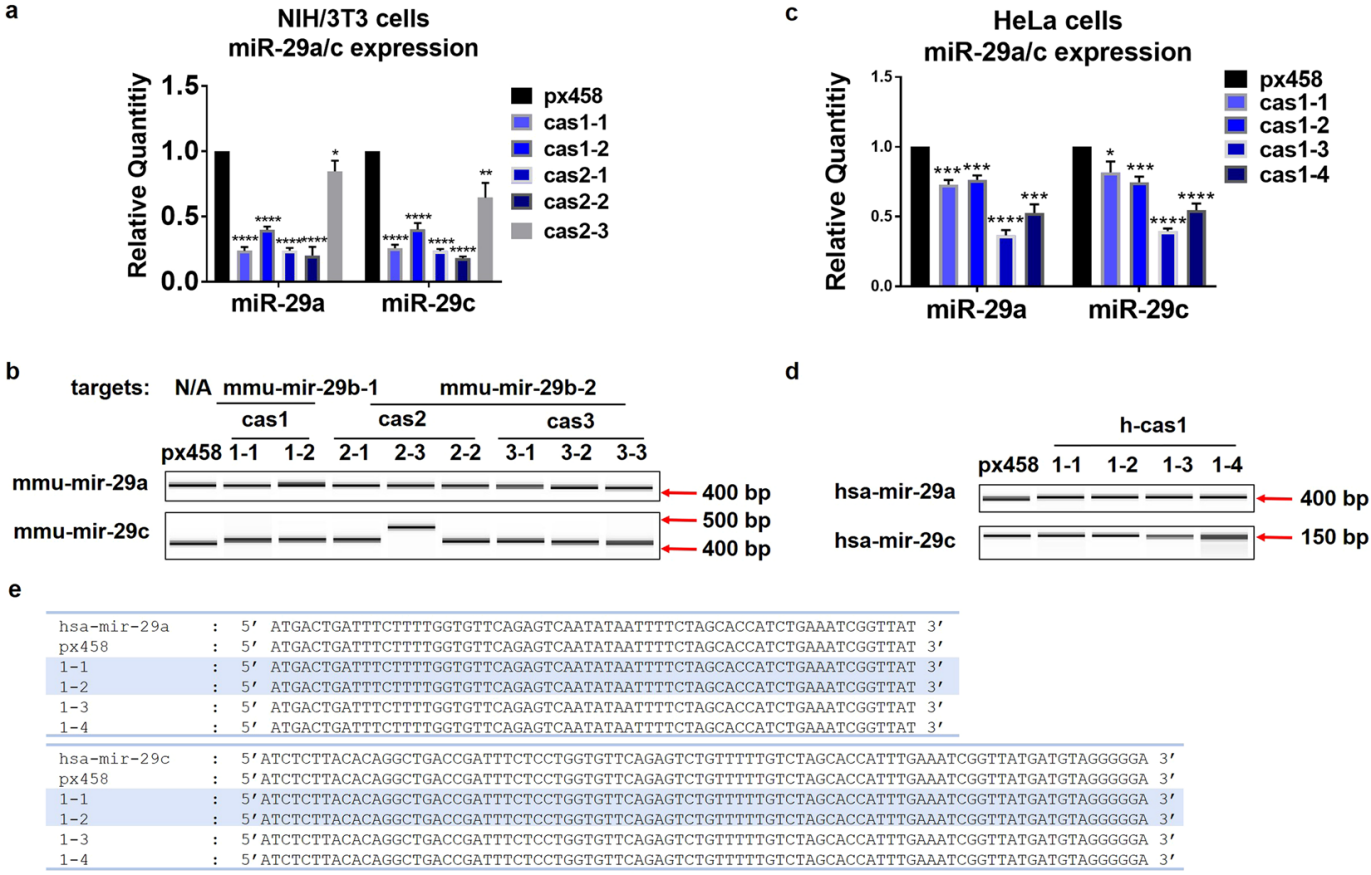
miR-29b knockdown decreased mature miR-29a/c expression levels, however did not change miR-29a/c genome sequences. (**a**) qRT-PCR assay showed that the mature levels of miR-29a and miR-29c were significantly downregulated in clones cas1-1, cas1-2, cas2-1 and cas2-2 compared to px458. The data shown represent the mean change with standard deviation from three times sample collection. Statistical analysis was performed using student t-test. *p < 0.05, **p < 0.01, ****p < 0.0001. (**b**) Surveyor assay detecting the changes on the nucleotide sequences of mmu-mir-29a and mmu-mir-29c were performed using primers designed spanning the genes locus. mmu-mir-29a displayed no changes among clones; mmu-mir-29c showed an increase in clone cas2-3. (**c**) qRT-PCR detected the downregulation of mature miR-29a and miR-29c in all four HeLa clones. Data shown represent the mean change with standard deviation from three experiments. Statistical analysis was performed using student t-test. *p < 0.05, ***p < 0.001, ****p < 0.0001. (**d**) Surveyor assay showed that no significant cleavage occurred on hsa-mir-29a/c sequences. (**e**) Sanger sequencing displayed no nucleotide changes on the genome locus of hsa-mir-29a and hsa-mir-29c. Surveyor assay products were visualized on Bioanalyser equipment using DNA 1000 chip. Full-length Bioanalyser images for (**b**,**d**) are presented in Supplementary Fig. 6.

To examine the impact of CRISPR/Cas9 editing on miR-29b family members, levels of mature miR-29a and miR-29c and their originating genes were detected using qRT-PCR and Surveyor assay. In NIH/3T3 cells, mature miR-29a and miR-29c levels were significantly decreased in clones cas1-1, cas1-2, cas2-1, cas2-2 and cas2-3 (Fig. 4a). The decrease of miR-29a and miR-29c in clone cas2-3 was to a much less extent compared to other clones (Fig. 4a); this is in accordance with the insignificant miR-29b knockdown in clone cas2–3 (Fig. 1b). No size changes were observed in mmu-mir-29a gene (Fig. 4b), implying that mature miR-29a level changes were not due to the disruption of mmu-mir-29a nucleotide sequences. There was a mild increase in the size of mmu-mir-29c gene in clone cas2-3, which displayed no downregulation of mature miR-29b (Fig. 4a), indicating the potential off-target effect of gRNA m-cas2 on mmu-mir-29c gene; no changes on mmu-mir-29c gene were observed in the other clones (Fig. 4b).

In HeLa cell clones, mature miR-29a and miR-29c were significantly decreased compared to px458 in the HeLa clones (Fig. 4c); no nucleotides changes were observed in has-mir-29b-2, has-mir-29a and has-mir-29c genes in all clones (Fig. 4d). DNA sequencing detected no changes on the nucleotides of mir-29a and mir-29c from the genomic DNA products of cell clones (Fig. 4E), demonstrating that gRNA h-cas1 targeting at hsa-mir-29b-1 gene is highly specific and effective at downregulating miR-29b.

**Figure 5:**
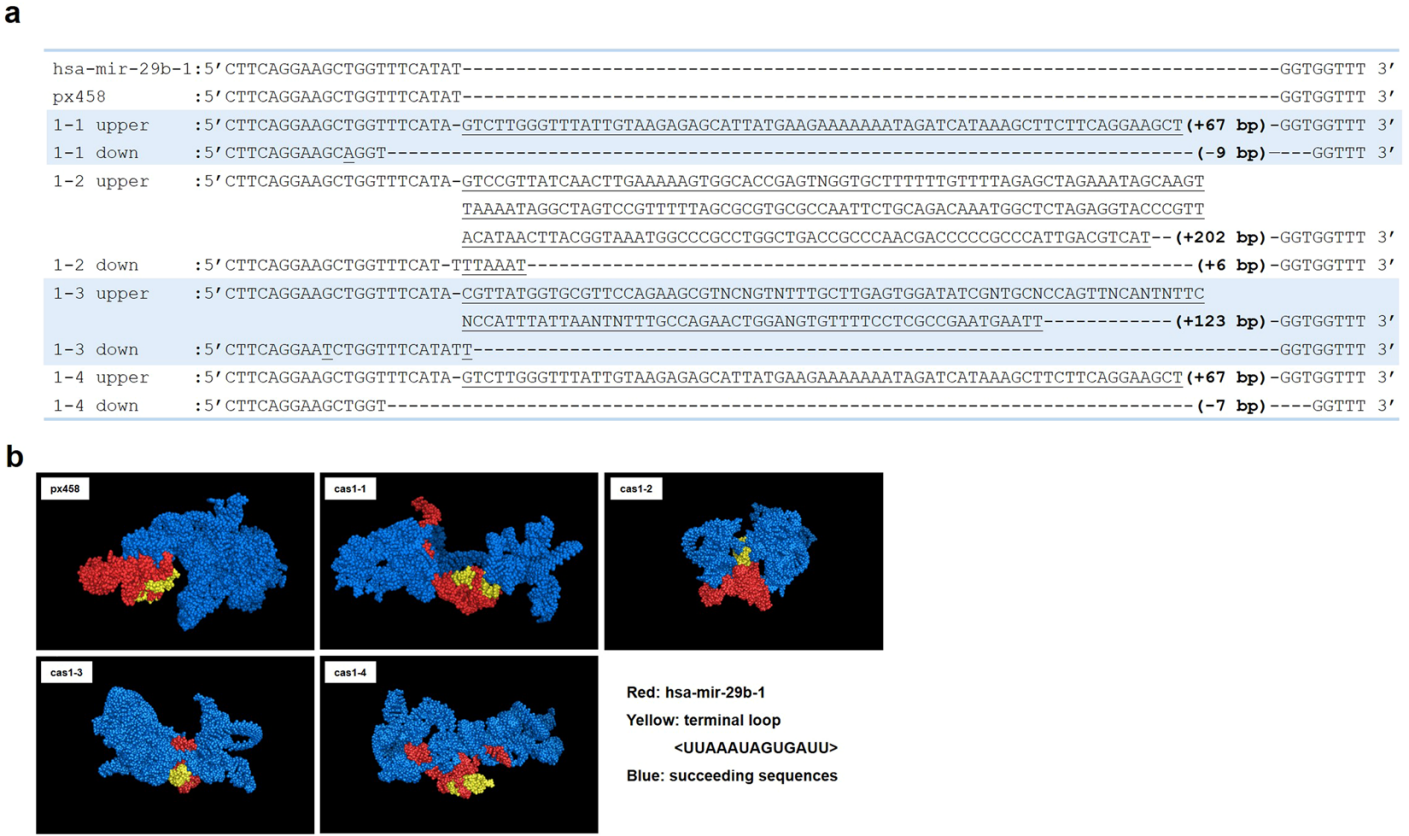
miR-29b editing changes the tertiary structures of mir-29b-1 succeeding sequences. (**a**) Sanger sequencing data displayed the nucleotide changes in all the clones. The upper bands of the four clones displayed insertions of 67 bp, 202 bp, 123 bp and 67 bp, respectively. The lower bands displayed minor insertions or deletions. Only partial sequences of hsa-mir-29b-1 were displayed. (**b**) Computational stimulation of the 3D structure of hsa-mir-29b-1 and succeeding sequences in HeLa cell clones with minor insertions or deletions following miR-29b editing. The 3D structure was generated by vsfold5 as input to RNAComposer, pdb files was visualised by PyMOL software.

To elucidate the mechanisms behind decreased mature miR-29a/c levels but intact nucleotide sequences, it was speculated that the tertiary structure of miR-29a/b-1 and miR-29b-2/c clusters may have been affected by mir-29b-1/b-2 sequence changes. DNA Sanger sequencing showed that HeLa cell clones cas1-1, cas1-2, cas1-3 and cas1-4 displayed nucleotide insertions of 67 base pairs, 202 bp, 123 bp and 67 bp, respectively (Fig. 5a). Clone cas1-1 also displayed deletions of 9 bp, cas1-2 showed insertions of 6 bp, cas1-3 displayed mutations at 2 nucleotides with no length changes, and cas1-4 obtained a 7 bp deletion (Fig. 5a). The computer stimulation of the tertiary structures of hsa-mir-29b-1 and succeeding sequences were performed on the small deletions, insertions or mutations of the four HeLa clones (Fig. 5b). Due to the software limitations, only 450 nucleotides encompassing hsa-mir-29b-1 or mutations and the succeeding sequences towards mir-29c were calculated for 3D structures (mir-29b-1 to mir-29c expands over 750 bp). Significant tertiary structure changes were observed in cas1-1, cas1-2, cas1-3 and cas1-4 with minor mutations, compared to the unedited mir-29b-1 in px458 control (Fig. 5b).

Since mir-29b-1 and mir-29a genes are only separated by a few hundred nucleotides, and reside as a miRNA cluster on the chromosome 6 and share the same promoters^21^, disruptions in the nucleotides sequences of mir-29b-1 genes may cause changes in the tertiary structures of mir-29a genes^36^; this may also affect the promoter functions regulating miRNA transcriptions^37^, which potentially contribute to the downregulation of mature miR-29a expression levels; the same mechanism applies to mir-29b-2 and mir-29c gene cluster, thus affecting the miRNA gene transcription and mature miR-29a and miR-29c generation.

### miR-29b shows specific targets and regulatory pathways in two cell types

#### Differentially expressed genes (DEGs) induced by miR-29b knockdown in NIH/3T3 and HeLa cells

The transcriptome profiles in cell clones following miR-29b editing were compared between NIH/3T3 and HeLa cells, aiming to identify the regulation patterns of miR-29b in these two distinct cell lines, and the novel regulation targets and pathways. The DEGs identified do not overlap with the predicted off-target genes list in Tables 1, 2, and 3 thus represent the miR-29b knockdown induced changes, distinguishing from the CRISPR/Cas9 editing effect.

Since each cell clone population was originated from one single cell screened and picked using FACS cell sorting, the individual cells within one sample are homogenous and the DEG profiles are the average effect of many identical cells. Four technical replicates for each cell clone sample were used to ensure the accurate presentation of the sample. Additionally, the DEG profiles were overlapped among all cell clones, which serve as biological replicates to each other, to ensure the *bona fide* miR-29b knockdown effect in both cell lines for target and pathway analysis.

**Figure 6:**
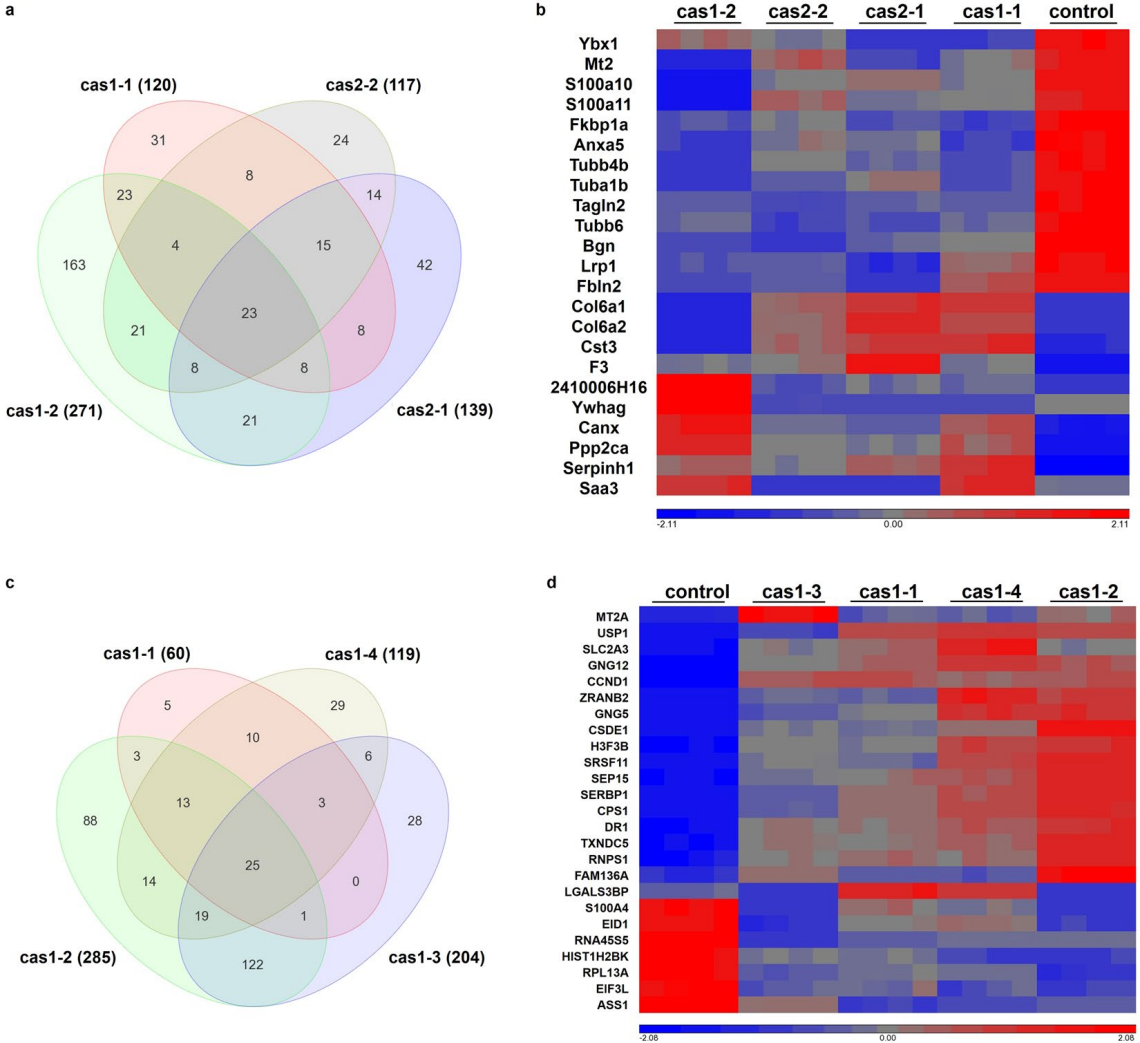
Differential gene expressions following miR-29b knockdown in NIH/3T3 and HeLa clones. DGEs assay was performed using Partek Genome Suite platform, with p value set less than 0.05. (**a**) The venn diagram displayed the numbers of DGEs in NIH/3T3 clones compared to px458. 23 genes were overlapped from all clones. (**b**) Heatmap showing the overlapped genes expressions in NIH/3T3 cell clones. (**c**) The venn diagram displayed the numbers of DGEs in HeLa clones compared to px458. 25 genes were overlapped from all clones. (**d**) Heatmap showing the overlapped genes expression across all HeLa cell clones.

In NIH/3T3 cells, there are 120, 271, 139 and 117 genes with over 1.5-fold changes detected in clones cas1-1, cas1-2, cas2-1 and cas2-2, respectively, compared to px458 (Fig. 6a). 23 genes were found to be dysregulated among all clones compared to px458, including upregulated Col6a1, Col6a2, Cst3, F3, 2410006H16, Ywhag, Canx, Ppp2ca, Serpinh1 and Saa3, and downregulated Ybx1, Mt2, S100a10, S100a11, Fkbp1a, Anxa5, Tubb4b, Tuba1b, Tagln2, Tubb6, Bgn, Lrp1, and Fbln2 (Fig. 6a,b).

The miRNA target prediction database www.microrna.org was used to assess whether these DEGs were targeted by miR-29b in their 3′ UTRs; mirSVR score represents the effect of a miRNA on target downregulation, combining both non-canonical and non-conservative binding sites, with a lower value represents a strong repression from miRNA on the target^38^. PhastCons score is the conservative score for the target and binding sites among species^39^. Among these genes, upregulated Canx, Ppp2ca, 2410006H16, Cst3, Col6a1, and Col6a2 were predicted to have potential binding sites for miR-29b in their 3′ UTRs (Table 4); downregulated Fkbp1a and Ybx1 were also on the list (Table 4), suggesting that miR-29b may function to activate the expression of these two genes.

**Table 4:** The DEGs potentially targeted by miR-29b in NIH/3T3 and HeLa cells.

gene symbol	mirSVR score	PhastCons score
**NIH/3T3 Cells**		
Canx	−0.4050	0.6267
Ybx1	−0.3521	0.5000
Ppp2ca	−0.3478	0.6607
Col6a2	−0.2156	0.4103
Fkbp1a	−0.0413	0.5768
Col6a1	−0.0049	0.5009
2410006H16	−0.0031	0.5005
Cst3	−0.0024	0.4762
**HeLa Cells**		
FAM136A	−1.0536	0.5906
CPS1	−1.0138	0.5915
SLC2A3	−0.3123	0.5347
SERBP1	−0.2185	0.6763
GNG12	−0.1842	0.6050
CSDE1	−0.1433	0.7753
DR1	−0.0082	0.5798
CCND1	0.0000	0.3450

In HeLa cell clones, cas1-1, cas1-2, cas1-3 and cas1-4 were shown to have 60, 285, 204 and 119 DEGs that have greater than 1.5-fold changes, respectively (Fig. 6c). There are 25 overlapping genes from all four clone groups, including upregulated MT2A, USP1, SCL2A3, GNG12, CCND1, ZRANB2, GNG5, CSDE1, R3F3B, SRSF11, SEP15, SERBP1, CPS1, DR1, TXNDC5, RNPS1, FAM136A, LGALS3BP, and downregulated S100A4, EID1, RNA45S5, HIST1H2BK, RPL13A, EIF3L, ASS1 (Fig. 6c,d). FAM136A, CPS1, SLC2A3, SERBP1, GNG12, CSDE1, DR1, and CCND1 are predicted to having binding sites for miR-29b in their 3′ UTRs (Table 4), implying the potential direct suppression effect of miR-29b on these genes.

#### Cell type specific targets and pathways induced by miR-29b knockdown

ConsensusPathDB database^40^ was used to analyse the pathways and gene ontologies (GOs) of differentially expressed genes (DEGs)^40^. In NIH/3T3 cells, the DEGs were found to be enriched in pathways such as extracellular matrix (ECM) organization, PI3K-Akt signalling pathway, phagosome, collagen formation, gap junction, chaperonin-mediated protein folding, NF-kB signalling pathway, Hippo pathway, Oocyte meiosis, mitotic phases transition, calcium regulation, PDGF and Wnt signalling pathways (Table 5). The gene ontology analysis (Table 5) is in accordance with the pathway analysis, with the GO terms being extracellular exosomes, macromolecular complex assembly, regulation of catalytic activity, endoplasmic reticulum, hemostasis and response to metal ion (Table 5).

**Table 5:** Pathway and gene ontology (GO) analysis of the DEGs in NIH/3T3 cells.

pathways	gene symbols
Extracellular matrix organization	Col6a1 Col6a2 Serpinh1 Fbln2 Bgn
PI3K-Akt signalling pathway	Col6a1 Col6a2 Ppp2ca Ywhag
Phagosome	Tuba1b Tubb6 Tubb4b Canx
Collagen formation	Col6a1 Col6a2 Serpinh1
Gap junction	Tuba1b Tubb6 Tubb4b
Chaperonin-mediated protein folding	Tuba1b Tubb6
TNFα NF-kB signalling pathway	Ywhag Ppp2ca
Hippo signalling pathway	Ywhag Ppp2ca
Oocyte meiosis	Ywhag Ppp2ca
Mitotic G2-G2/M phases transition	Ywhag Ppp2ca
Calcium regulation	Ywhag Fkbp1a
PDGF signalling pathway	Col6a1 Col6a2
Wnt signalling pathway	Lrp1 Ppp2ca
**gene ontology term**	**gene symbols**
Extracellular exosomes	Anxa5 Col6a2 Tuba1b Fkbp1a Bgn Ywhag Cst3 F3 Ppp2ca Serpinh1 Col6a1 Tubb6 Fbln2 Tubb4b S100a10, Tagln2 and Ybx1
Neuronal cell bodies	Anxa5 Tubb4b Tuba1b Fkbp1a Ywhag Cst3 Lrp1 Canx Ppp2ca
Macromolecular complex assembly	Tubb6 Col6a1 Col6a2 Ppp2ca Tubb4b Tuba1b
Regulation of catalytic activity	Serpinh1 F3 Fkbp1a Lrp1 Ppp2ca
Endoplasmic reticulum	Canx Serpinh1 Cst3 Fkbp1a
Hemostasis	Anxa5 F3
Response to metal ion	Mt2 Fkbp1a

In HeLa cell clones, the DEGs were found to be involved in protein metabolism, urea cycle, chaperonin-mediated protein folding, G protein signalling, calcium pathway, copper homeostasis, chromatin organization, alcoholism, Wnt signalling, PI3K-Akt signalling, C-Myc transcriptional repression, cellular senescence, amyloid fiber formation, DNA methylation, meiosis, hemostasis, and mRNA 3′ UTR mediated regulation (Table 6). Through GO analysis, cellular macromolecular complex assembly, RNA binding, response to bacterium, zinc ion, glucagon, hormone and cytokines were identified (Table 6).

**Table 6:** Pathway and gene ontology (GO) analysis of the DEGs in HeLa cells.

pathways	gene symbols
Metabolism of proteins	H3F3B HIST1H2BK GNG5 GNG12 RPL13A EIF3L
Metabolism of amino acids and derivatives	ASS1 CPS1 RPL13A
Urea cycle	ASS1 CPS1
Chaperonin-mediated protein folding	GNG5 GNG12
G Protein signalling pathways	GNG5 GNG12
Calcium pathway	GNG5 GNG12
Copper homeostasis	MT2A CCND1
Chromatin organization	HIST1H2BK CCND1 DR1
Alcoholism	GNG5 HIST1H2BK H3F3B GNG12
Wnt signalling pathway	GNG5 HIST1H2BK H3F3B GNG12
Pathways in cancer	CCND1 GNG5 GNG12
PI3K-Akt signalling pathway	CCND1 GNG5 GNG12
Focal Adhesion-PI3K-Akt-mTOR-signalling	SLC2A3 GNG5 GNG12
Targets of C-MYC transcriptional repression	CSDE1 CCND1
Cellular senescence	H3F3B HIST1H2BK
Oxidative stress induced senescence	H3F3B HIST1H2BK
Amyloid fiber formation	H3F3B HIST1H2BK
DNA methylation	H3F3B HIST1H2BK
Meiosis	H3F3B HIST1H2BK
Hemostasis	H3F3B LGALS3BP GNG5 GNG12
Cell Cycle, Mitotic	HIST1H2BK CCND1 LGALS3BP
mRNA 3′ end processing	RNPS1 SRSF11
3′ UTR-mediated translational regulation	RPL13A EIF3L
**gene ontology term**	**gene symbols**
Cellular macromolecular complex assembly	H3F3B EIF3L HIST1H2BK RPL13A
poly(A) RNA binding	ASS1 S100A4 CSDE1 SRSF11 ZEANB2 RNPS1 EIF3L RPL13A SERBP1
Response to bacterium	ASS1 CPS1 GNG12 HIST1H2BK
Response to zinc ion	ASS1 CPS1 MT2A
Response to glucagon	ASS1 CPS1 GNG5 GNG12
Response to peptide hormone stimulus	ASS1 CPS1 GNG5 GNG12
Response to interferon-gamma	ASS1 MT2A TPL13A

When comparing these two cell lines, it is noticeable that both cell lines have common GO terms including macromolecular complex assembly, and response to stimulus (Tables 5 and 6). Both cell lines share common pathways and functions associated with miR-29b knockdown, including PI3K-Akt pathway, Wnt signalling pathway, cell cycle regulation, chaperonin-mediated protein folding, and calcium regulation (Table 3a,c). Hemostasis regulation was identified in GO analysis of NIH/3T3 cell clones and in pathway analysis of HeLa cell clones (Tables 5 and 6). Although these two cell lines share similar pathways and gene ontologies induced by miR-29b knockdown, the DEGs involved in these pathways and GO terms are distinct.

In NIH/3T3 cells, Tubb6, Col6a1, Col6a2, Ppp2ca, Tubb4b and Tuba1b are implicated in macromolecular complex assembly (Table 5), while in HeLa cells, H3F3B, EIF3L, HIST1H2BK and RPL13A are involved in the same GO term (Table 6). In NIH/3T3 cells, Mt2 and Fkbp1a are shown to be involved in response to metal ion (Table 5). In HeLa cells, ASS1, CPS1, GNG12, HIST1H2BK, MT2A, GNG5, and TPL13A are implicated in response to bacterium, zinc, glucagon, hormone, and interferon γ (Table 6). miR-29b has been reported to sensitize cells to apoptosis induced by serum starvation, hypoxia or chemotherapeutic drugs through targeting MCL-1 and BCL-2^41^. These DEGs involved in cellular response to various stimuli may serve as the connecting bridges through which miR-29b plays functional roles in apoptosis, growth or inflammation.

DEGs involved in the common PI3K-Akt pathway include Col6a1, Col6a2, Ppp2ca and Ywhag in NIH/3T3 cells (Table 5), and CCND1, GNG5 and GNG12 in HeLa cells (Table 6). Lrp1 and Ppp2ca in NIH/3T3 cells and GNG5, GNG12, HIST1H2BK and H3F3B in HeLa cells are involved in Wnt signalling pathway (Tables 5 and 6). Newly identified miR-29b targets in cell cycle regulations include Ywhag, Ppp2ca in NIH/3T3 cells, and H3F3B, HIST1H2BK, CCND1 and LGALS3BP in HeLa cells (Tables 5 and 6). Tuba1b and Tubb6 in NIH/3T3 cells, and GNG5, GNG12 in HeLa cells are implicated in chaperonin-mediated protein folding. Ywhag and Fkbp1a in NIH/3T3 cells and GNG5 and GNG12 in HeLa cells are involved in calcium pathways. Hemostasis regulation was identified NIH/3T3 cell clones involving Anxa5 and F3, and in HeLa cell clones involving H3F3B, LGALS3BP, GNG5 and GNG12. The only gene that was dysregulated in both cell lines is Mt2 (MT2A in human species), which was implicated in response to metal ions in both cell lines (Tables 5 and 6).

These two cell lines also exhibited cell type specific pathways and functions associated with miR-29b knockdown. Most DEGs in NIH/3T3 cell clones are involved in extracellular matrix organization, extracellular exosomes and neuronal cell bodies (Table 5), while DEGs in HeLa cell clones are enriched in protein metabolism, cancer associated pathways, cellular senescence, and RNA binding and processing (Table 6). miR-29b has been reported to play functional roles in fibrosis and tumorigenesis^16,42^. The novel miR-29b targets identified in extracellular vesicles regulations and neuronal cell bodies in NIH/3T3 cells, and in cellular senescence and RNA processing/regulation in HeLa cells, illustrated the cell type specific regulation network of miR-29b in these two cell lines.

## Discussion

Despite the growing interest in studying miRNAs of their roles as disease diagnostic biomarkers and as regulators of disease associated proteins or/and genes, the exact miRNA regulatory mechanisms in different cellular environments and species are still not clear. In this study, CRISPR/Cas9 gene editing was used to effectively knockdown miR-29b in two different cell lines, NIH/3T3 and HeLa cells. It was revealed that CRISPR/Cas9 editing is highly specific at recognizing and cleaving target genome loci; the specific gRNA targeting revealed mir-29b-1 to be the major source of generating mature miR-29b rather than mir-29b-2 in both cell lines. The off-target effect of CRISPR/Cas9 editing is minimum on transcriptome level. However, the editing did induce the expression level changes of miR-29 family members without affecting their nucleotide sequences, potentially via changing the tertiary structures of miRNA gene surroundings and affecting the transcription of mir-29 genes. Transcriptome profiling of the cell clones identified common and cell type specific regulation pathways associated with miR-29b knockdown and revealed distinct novel targets of miR-29b in NIH/3T3 and HeLa cells.

In miR-29b editing, gRNAs h-cas1 and m-cas1 share the same nucleotide sequences, and both induced sufficient miR-29b knockdown. Distinct nucleotide changes on miR-29b-1 genes were observed using the same gRNA, due to the non-specific NHEJ repair pathway^29^, suggesting that the CRISPR/Cas9-NHEJ editing is effective at disrupting target miRNA expression, but the nucleotide changes are introduced in a random way. More than one cell clones should be tested to identify the clones with the least off-target effect and good knockdown efficiency as further research models.

A large part of the predicted off-targets for miR-29b gRNAs were also potentially targeted by miR-29a*, b*, or/and c*. Although miRNA targeting mechanisms are different to CRISPR/Cas9 editing, it is possible that guide RNAs may target miRNA targets. The gRNAs in this study were designed to target the 5′ end of the miR-29b gene sequences, which overlap with the sequences that later form mature miR-29b*^13^. The sequences of miR-29a and miR-29c genes also share similarities with miR-29b genes^13^. Consequentially, miR-29a* or/and miR-29c* may target some predicted off-targets of miR-29b gRNAs.

Off-target cleavage activities are closely related to the numbers and positions of the mismatches between gRNAs and off-target sites, wherein <=2 mismatches can result in high cleavage activities and >=3 mismatches cause extremely low cleavage activities^43,44^. While most predicted genes have >=3 mismatches and scores approaching 0, mmu-mir-29b-2 in Table 1, mmu-mir-29b-1 in Table 2, and hsa-mir-29b-2 in Table 3 are the only 3 genes with <=2 mismatches and high scores of 2.3. These high-ranking potential off-targeted genes with <=2 mismatches were examined using Surveyor assays (Fig. 2). Methods such as GUIDE-Seq or CIRCLE-Seq have been developed to evaluate the genome nuclease cleavage activities^45,46^, ensuring the precise examination of off-target effects for future studies.

miR-29b is transcribed from two genome loci; each miR-29b gRNA was designed to target only one gene locus, leaving the other miR-29b gene site with high risk of being off-targeted, due to the sequence similarities of the two miR-29b coding genes. Surveyor assay and DNA sequencing analysing the sizes and nucleotide sequences of the ‘off-target’ miR-29b gene revealed no changes induced by gRNAs h-cas1 and m-cas2 in HeLa cells and NIH/3T3 cells respectively. Through RNA sequencing the only ‘verified’ off-targets include downregulated Fkbp1a in NIH/3T3 cells and upregulated H3F3B and JAK1 in HeLa cells. However, they are more likely to be the ‘on-target’ rather than ‘off-target’ genes due to the potential binding sites identified in their 3′ UTRs for miR-29b^39^. Overall, with proper gRNA design, CRISPR/Cas9 mediated miR-29b editing is highly accurate and specific at recognizing target genome loci.

Despite the high specificity of miR-29b editing, the tertiary structures of miR-29b gene surroundings are likely to be affected, thus inducing expression level changes of neighbouring miRNAs or genes, in this case, the miR-29a and miR-29c genes. It has been reported in 2017 that CRISPR/Cas9 mediated miR-195 editing led to a significant decrease in the expression of miR-497a in the miR-497~195 cluster, with no gene editing detected in miR-497a gene locus, while tertiary structure prediction revealed an altered 3D structure of the miRNA cluster^47^. Additionally, miRNA clusters are likely to share the same promoters for their transcriptional regulation^7,21^, while disruption on one miRNA of the cluster may cause changes on the sequences or structures of their promoters and thus affect the transcription of other miRNAs.

The study also revealed that miRNAs can be involved in highly selective gene regulation. After overlapping the DEG profiles across multiple cell clones, a specific set of 23 and 25 DEGs were identified in NIH/3T3 and HeLa cells respectively. These are extremely small sets of genes comparing to what we get after “normal gene” editing. Rather, the small set of DEG may be a reflection of the specificity of miR-29b which would result in highly relevant pathways upon gene enrichment analysis. The fine-tuning characteristic of miRNAs has been reported but was only investigated in specific pathways^48^. Our study addressed this question by showing the effect of miR-29b stable knockdown in the whole transcriptome. The DEG profiles identified are small and distinct in these two cell lines, yet some common pathways were identified, validating the functions of miR-29b regardless of its actual targets in different cell lines.

miR-29b knockdown in NIH/3T3 and HeLa cells induced DEGs involved in common pathways and functions, reflecting the conservative roles of miR-29b in regulating cell cycle, Wnt and PI3K-Akt signalling pathways, and macromolecular complex assemble. However, the DEGs involved in the same pathways in two cell lines are distinct. Ywhag and Ppp2ca were identified as cell cycle regulators targeted by miR-29b in NIH/3T3 cells, however in HeLa cells, HIST1H2BK, CCND1 and LGALS3BP targeted by miR-29b. This phenomenon indicates the conservative roles of miR-29b and its ability to adapt to distinct cellular environments via targeting a different set of gene networks.

miR-29b knockdown also revealed cell type specific features of its functions in these two cell lines. In NIH/3T3 cells, ECM regulation enriched most DEGs including Col6a1, Col6a2, Serpinh1, Fbln2 and Bgn, among which Col6a1 and Col6a2 have predicted binding sites with miR-29b (Table 4). The roles of miR-29b in ECM mediated fibrosis have been widely depicted. TGF-β signalling mediated miR-29b downregulation has been shown to mediate fibrotic pathologies via upregulating collagen proteins COL1A1, COL5A3 and COL4A2^49^, which belong to the same family with Col6a1 and Col6a2 encoded proteins. Fbln2 encodes for Fibulin 2 protein, which is distributed abundantly in elastic tissues and can interact with various extracellular ligands in calcium-dependent way^50^. miR-29b has been shown to target fibrillins and elastin in ECM regulations^16^, implying Fbln2 as the novel regulator in miR-29b-fibrillins/elastin regulation network. Bgn is a member of the small leucine rich proteoglycan family of proteins, and was implicated in collagen fibril assembly^51^. Several DEGs are implicated in neuronal bodies in NIH/3T3 cells, and a few of them have reported roles in neurodegeneration. Lrp1 has been reported to participate in APP and Aβ clearance in AD^52,53^. The mutation in Cst3 (Cystatin C) has been revealed to be associated with cerebral amyloid angiopathy and Aβ pathway^54^. Saa3, encoding for serum amyloid A3, is involved in the suppression of LPS-induced tau hyperphosphorylation^55^, suggesting the important roles of Lrp1, Cst3 and Saa3 in AD pathogenesis. Some DEGs are found to be related to prion disease, including Ywhag, Mt2, Serpinh1 and Cst3. Ywhag has been reported to be a diagnostic marker for sporadic CJD^56,57^. Mt2 (Metallothionein 2A) receptor can enhance the synaptic transmission by activating Akt signalling^58^; it is involved in the response to metal ions and its polymorphism has been involved in inflammatory response in diseases such as type 2 diabetes and carotid artery stenosis in elderly people^59–61^, increased Mt2 expression was also observed in prion-infected hamster brains^62^. Multiple DEGs in NIH/3T3 cells are identified in extracellular exosomes^63,64^ (Table 5); which has important implications for the potential role of miR-29b in exosome mediated cargo delivery between cells and tissues.

In HeLa cells, the most prominent function of miR-29b is its involvement in tumorigenesis. Dysfunction of cell cycle regulation, epigenetic regulation and protein metabolism are molecular events that are closely associated with tumorigenesis^65–69^. One novel miR-29b target identified is CCND1, with predicted binding sites with miR-29b (Table 4); it encodes for cyclin D1, whose activity is required for G1/S transition during cell cycle^70^. miR-29b has been reported to decrease in MCL cell models, the causative event of which is associated with overexpression of cyclin D1 and subsequent activation of CDK6 by miR-29b^18^. Cyclin D1 was also involved in IL-7 signalling pathway and p53 signalling pathways^71,72^, and mutations or dysregulation of cyclin D1 alter cell cycle progression, resulting in a variety of tumors^73,74^. H3F3B and HIST1H2BK are novel targets identified in epigenetic regulation (Table 6). Mutation of H3F3B has been reported as a diagnostic biomarker for giant cell tumor of bone and chondroblastoma^75^. The novel targets mediated HeLa cell specific functions of miR-29b were also demonstrated by the miR-29b knockdown induced cellular senescence and RNA processing pathways (Tables 5 and 6).

Common pathways involving different targets were identified in fibroblasts and cancer cells, such as cell cycle, Wnt and PI3K-Akt signalling pathways and macromolecular complex assemble. The differences observed between miR-29b targets and pathway enrichment analysis in the two cell lines may reflect the cell types and their specific characteristics. Nevertheless, the differences driven by the species are not entirely clear. As a future perspective, it is worth to investigate the specificity of the target driven by cell type or species by comparing more cell lines from each species and multiple cell types from one species.

Overall, stable miR-29b knockdown using CRISPR/Cas9 editing was successfully done in selected human and mouse cell lines, despite the short length of miRNA genes. The CRISPR/Cas9 editing of miR-29b revealed new mechanisms of the biogenesis of miR-29b - that mir-29b-1 is the major source to generate mature miR-29b other than mir-29b-2. The miR-29b editing also shed light on the novel miRNA cluster regulations, by depicting the tertiary structure changes of mir-29b-1 surroundings. The specific functions reflecting cell characteristics and novel targets of miR-29b were revealed for the first time between two cell lines. Novel miR-29b targets associated with ECM organization, extracellular vesicles and neurodegenerative disorders in mouse NIH/3T3 cell lines and the cellular senescence, RNA processing and regulation in human HeLa cells, provided critical references for the selective targeting mechanisms of miRNAs.

## Materials and Methods

### Maintenance of cells

Cell culture was performed in Class 2 BioSafety Cabinet under sterile conditions in a Physical Containment Level 2 (PC2) facility. The cells were cultured in Dulbecco’s Modified Eagle Medium (DMEM) supplemented with 10% (v/v) heat inactivated Fetal Bovine Serum (FBS), 100 units of Penicillin-Streptomycin (10,000 U/ml), and 1x GlutaMAX, in tissue culture flasks in a humidified 5% (v/v) CO_2_ environment at 37 °C. Cells were passaged every two days with 1:10 splits.

### CRISPR/Cas9 plasmid construction

The guide RNA (gRNA) insertions were prepared through phosphorylating and annealing the top and bottom strands of the oligonucleotides, followed by ligation reaction to clone the insertions into px458 plasmid (pSpCas9-2A-GFP)^29^. PlasmidSafe treated plasmids were then transformed into One Shot Chemically Competent *E. coli* strain Stbl3 (ThermoFisher Scientific), and the colony growth was inspected the next day. For each construction, two or three colonies were picked to check for the correct insertion of the gRNAs.

### Cell transfection

Cells were plated at a density of 1.5 × 10^5^ cells per well (12-well plate) the day before transfection, reaching approximately 80% confluence prior to transfection. Reconstructed CRISPR/Cas9 plasmids were transfected into cells using Lipofectamine 3000 transfection reagents (ThermoFisher Scientific) according to manufacturer’s instructions.

### Microscope imaging

Cells transfected with the CRISPR/Cas9 plasmids were imaged using a Leica AF6000 widefield epi-fluorescence microscope (Leica Microsystems) using 10x and 20x objectives. Bright field images were taken at the same time with the same magnification power. The exposure time for all samples was set to be the same in each experiment. The images were annotated with micron scales and exported using Leica AF6000 imaging software.

### Fluorescence Activated Cell Sorting (FACS)

Cells transiently transfected with the reconstructed CRISPR/Cas9 plasmids were detached and washed in calcium and magnesium free DPBS. The un-transfected cells and cells transfected with px458 were used as the negative controls. The cells were then resuspended to a density of 0.5–1 × 10^7^ cells per ml in FACS buffer. EDTA was added to the cell suspension to a final concentration of 1–5 mM to prevent cells from clumping. To ensure that viable cells were collected, 1 µg/ml Propidium Iodide (PI) and 200 ng/ml DAPI were added to the cells just prior to cell sorting. Samples were filtered with 30–40 um strainers before being processed on the FACSAria equipment (BD Biosciences). Cell populations or single cells were collected into collection tubes or 96-well plates based on GFP signal strength.

### Surveyor assay

Genomic DNA was extracted from cell samples. Surveyor assay was performed using Surveyor Mutation Detection Kit (Integrated DNA Technologies) according to the manufacturer’s instructions. Briefly, PCR was performed on genomic DNA using Surveyor forward and reverse primers with the following cycling conditions: denature at 95 °C for 2 minutes; denature at 95 °C for 20 seconds, anneal at 60 °C for 20 seconds, and extend at 72 °C for 30 seconds; repeat the cycle for 30 times; extend at 72 °C for 3 minutes. The PCR products were electrophoresed on a 1% (w/v) agarose gel and purified using DNA clean-up procedure. The eluted PCR products were then annealed to form heteroduplex, which was then mixed with the Surveyor assay components for digestion at 42 °C for 30 minutes. Surveyor nuclease digestion products were visualized on Bioanalyser equipment using DNA 1000 chips (Agilent Technologies) containing interconnected microchannels for separation of nucleic acid fragment based on their sizes. Bioanalyser images and running information were exported as PDF files from Bioanalyser software.

### DNA sequencing

Primers U6-F was used for sequencing the CRISPR/Cas9 construct insertions. U6-F 5′-GAGGGCCTATTTCCCATGATTCC-3′. For purified PCR products, each fragment was washed in 70% ethanol and dried before being submitted for DNA sequencing. Primers used: h-miR-29b-1-F 5′-CTTCAGGAAGCTGGTTTC-3, h-miR-29b-1-R 5′-CTCCTAA AACAC TGATTT-3, h-miR-29b-2-F 5′-CTTCTGGAAGCTGGTTTC-3′, h-miR-29b-2-R 5′-CTC CTAA AACACTGATTT-3, h-miR-29a-F 5′-ATGACTGATTTCTTTTGGT-3′, h-miR-29a-R 5′-ATAACCGATTTCAGATGG-3′, h-miR-29c-F 5′-ATCTCTTACACAGGCTGA-3′. Sequencing results were viewed and aligned against the unedited sequences on the Geneious software.

### Quantitative Real-Time PCR (qRT-PCR)

qRT-PCR was performed using TaqMan MicroRNA Reverse Transcription Kit and miRNA primers (Applied Biosystems). miRNA primers used include miR-29b (Assay ID 000413), miR-29a (Assay ID 000412), miR-29c (Assay ID 000587) and U6 (Assay ID 001973). The PCR reaction was run on ViiA7 Real-Time PCR machine at the following conditions: 95 °C for 20 seconds; 40 cycles of 95 °C for 1 second, 60 °C for 20 seconds; 4 °C for holding. The relative miRNA fold changes against housekeeping control U6 were calculated using 2^−ΔΔCT^ method. The statistical significance tests were performed using unpaired Student’s t-tests against a standardised control.

### Computational analysis

Secondary structures of miR-29b gene and succeeding sequences were generated by vsfold5^76^. The RNAComposer^77^ was used to generate the pdb files through molecular RNA simulation from vsfold5 output. The pdb files were input to pyMOL to generate 3D images.

### mRNA library construction

Total RNA was extracted from the cell samples using RNeasy RNA extraction Kit and the concentration and quality were determined using Bioanalyzer RNA 6000 Kit (Agilent Technologies). The mRNA library construction of the samples was performed using NEBNext mRNA Library Prep Master Mix Set for Illumina, according to manufacturer’s instructions. RNA deep sequencing was performed with 2 × 150 bp paired end reading method on Illumina NextSeq platform (La Trobe University).

### RNA deep sequencing data analysis

The RNA sequencing data were analysed using Partek Flow and Partek Genomics Suite. Briefly, the adaptor sequences were trimmed from all reads generated and sequences were then aligned to the GRCh38 (Genome Reference Consortium Human Build 38)^78^ for the HeLa cell samples and GRCm38 (Genome Reference Consortium Mouse Build 38)^79^ for NIH/3T3 cell samples using Bowtie 2 algorithm. Aligned reads profiles were quantified and analysed for differential gene expressions^80^. ConsensusPathDB^40^ was used to analyse the pathways and gene ontologies (GOs) of differentially expressed genes (DEGs), wherein the gene set analysis was performed under over-representation analysis with significance score less than 0.05.

## Supplementary information

Supplementatry Information

## Data Availability

Microrna.org is an open source online tool for miRNA target predictions (http://www.microrn a.org/microrna/home.do). CRISPR/Cas9 gRNA design tool is available at (https://zlab.bio/guide-design-resources). ConsensusPathDB is an open source online database for pathway and gene ontology analysis (http://cpdb.molgen.mpg.de/). Vsfold5 is an open source online tool for nucleotide secondary structure prediction (http://www.rna.it-chiba.ac.jp/~vsfold/vsfold5/). RNAComposer is an open source online tool for generating pdb files (http://rnacomposer.cs.put.poznan.pl/).
