# Supplementary material for: Novel miR-29b target regulation patterns are revealed in two different cell lines
Source: Sci Rep. 2019 Nov 25;9:17449. doi: 10.1038/s41598-019-53868-x (PMC6877611; doi:10.1038/s41598-019-53868-x)
Supplement: Supplementary file 1 — Supplementatry Information [file 41598_2019_53868_MOESM1_ESM.docx]

**Novel miR-29b target regulation patterns are revealed in two different cell lines**

Wenting Zhao^1,2^, Lesley Cheng^1^, Camelia Quek^2^, Shayne A. Bellingham^2,*^ and Andrew F. Hill^1,*^

^1^ Department of Biochemistry and Genetics, La Trobe Institute for Molecular Science, La Trobe University, VIC, 3086, Australia

^2^ Department of Biochemistry and Molecular Biology, Bio21 Institute, The University of Melbourne, VIC, 3010, Australia

* To whom correspondence should be addressed. Tel: +61 3 9479 1224; Fax: +61 3 9479 2467; Email: [andrew.hill@latrobe.edu.au](mailto:andrew.hill@latrobe.edu.au)

Department of Biochemistry and Genetics

La Trobe Institute for Molecular Science

La Trobe University

Victoria 3086 Australia

**Supplementary Legends and Figures**

**Supplementary 1. Endogenous miR-29b is expressed at high levels in HeLa and NIH/3T3 cells.** qRT- PCR detection of endogenous miR-29b expression in human SH-SY5Y, HEK293T, HeLa cells, and in mouse N2a and NIH/3T3 cells. The expression of miR-29b is normalized to miRNA housekeeping control - U6, and compared with SH-SY5Y cells, which displayed the lowest expression of miR-29b. Data shown represent the mean change with standard deviation from three experiments. Statistical analysis was carried out using multiple Student’s t-tests, ** p<0.01, *** p<0.001, **** p<0.0001.

**Supplementary 2**. **No significant miR-29b expression changes were detected following transient transfection of CRISPR/Cas9 plasmids in NIH/3T3 cells.** CRISPR/Cas9 plasmids targeting miR-29b gene (cas1, cas2 and cas3) were transiently transfected into NIH/3T3 cells after optimizing transfection conditions. (a) About 15% of cells exhibited GFP fluorescence signal at 48 hrs post transfection in CRISPR transfected cells compared with untransfected blank cells; the bright field image showed the cell density when GFP fluorescence was detected. (b) The expressions of miR-29b from cas1/2/3 transfected cell groups displayed no significant change compared with px458 group. Data shown represent the mean change with stand deviation from three experiments. Statistical analysis was performed using student t-test, no significance was found.

**Supplementary 3. miR-29b is decreased in the cell groups with high GFP signals compared to cells with low GFP signals.** (a) FACS cell sorting was used to isolate cells transfected with cas1, cas2, cas3 and px458 plasmids into high GFP groups (P5 GFP+) and low GFP groups (P6 GFP-), based on the GFP fluorescence signal strength. (b) Real-time PCR demonstrated that miR-29b levels are significantly lower in high GFP groups in cas1 and cas2 compared to low GFP groups; high GFP group from cas3 also displayed lower level compared to px458. The data shown represent the mean change with standard deviation from three experiments. Statistical analysis was performed using multiple Student’s t-tests, * p<0.05, ** p<0.01.

**Supplementary 4. Isolation of single cells expressing h-cas1.** H-cas1 and px458 were transfected into HeLa cells. (a) showed the GFP fluorescence signal of px458, cas1 and untransfected control at 48 hrs post transfection. The bright field images showed the corresponding cell density. (b) Single cells from P4 group were collected into 96-well plate for clone culture using FACS cell sorting.

**Supplementary 5. gRNA mediated cleavage reveals mir-29b-1 as the main source of mature miR-29b.** (a) Surveyor assay detecting the mutations on miR-29b gene locus. mmu-mir-29b-1 was shown to have mutations in cas1-1, cas1-2, cas2-1 and cas2-2; mmu-mir-29b-2 displayed mutations in cas2-1, cas2-2, cas3-1, cas3-2 and cas3-3. (b) Surveyor assay showed mutations on hsa-mir-29b-1 and hsa-mir-29b-2 sequences in clone cas1-1, cas1-2, cas1-3 and cas1-4. Surveyor assay products were visualized on Bioanalyser equipment using DNA 1000 chip.

**Supplementary 6. Surveyor assay analysis of mir-29a and mir-29c.** (a) Surveyor assay detecting the changes on the nucleotide sequences of mmu-mir-29a and mmu-mir-29c were performed using primers designed spanning the genes locus. mmu-mir-29a displayed no changes among clones; mmu-mir-29c showed an increase in clone cas2-3. (b) Surveyor assay showed that no significant cleavage occurred on hsa-mir-29a/c sequences. Surveyor assay products were visualized on Bioanalyser equipment using DNA 1000 chip.

**Supplementary 1**

**Supplementary 2**

**
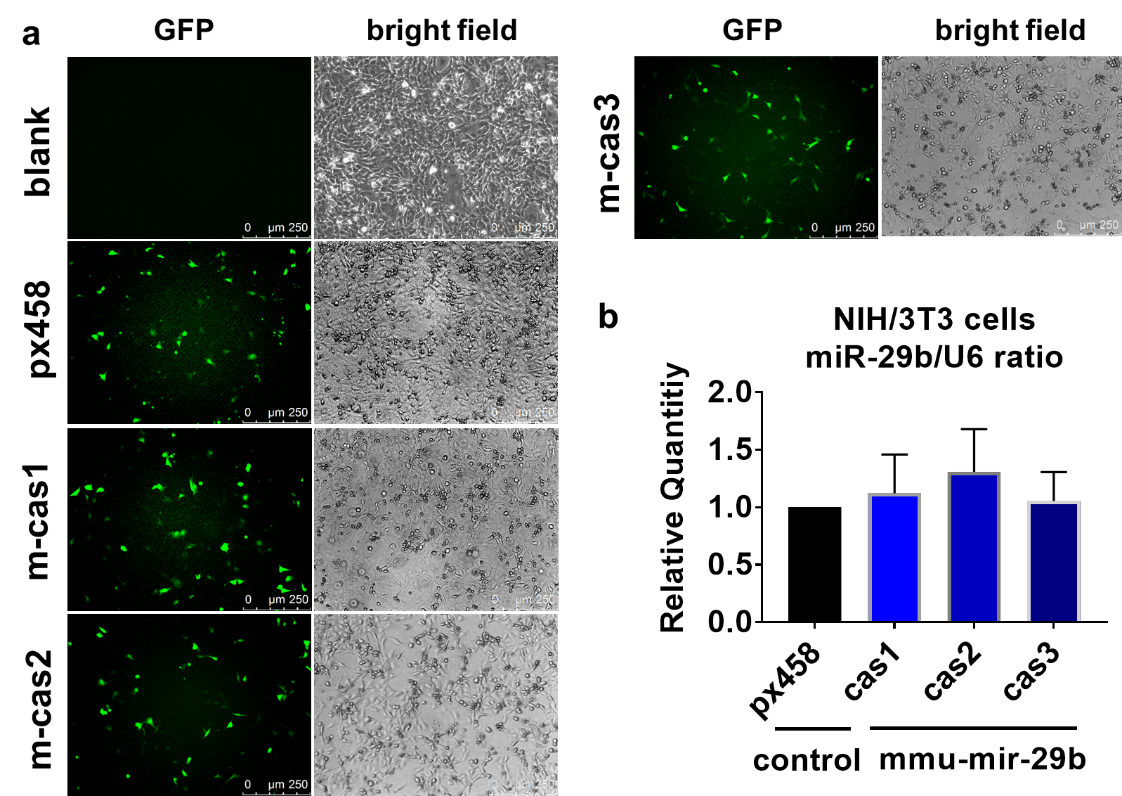
**

**Supplementary 3**

**
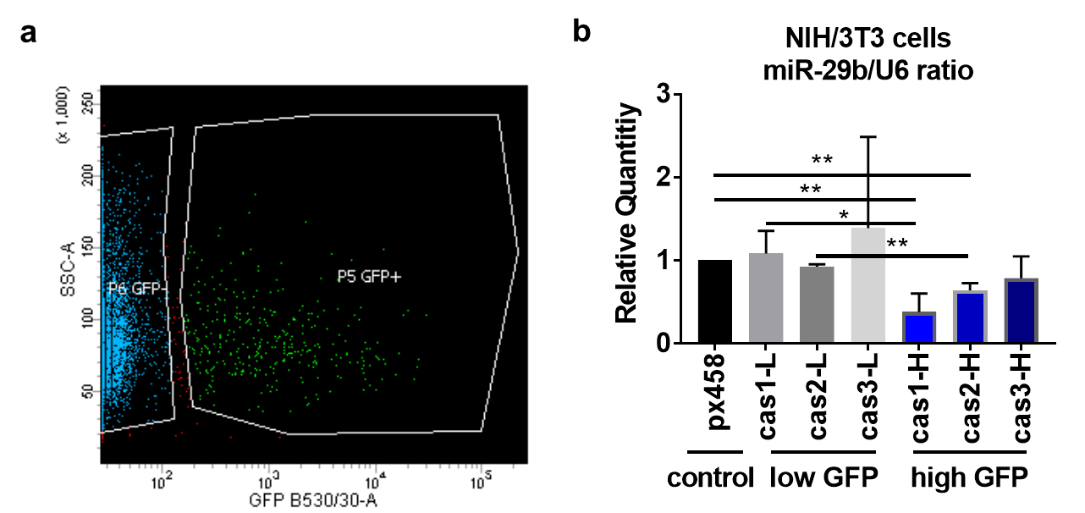
**

**Supplementary 4**

**
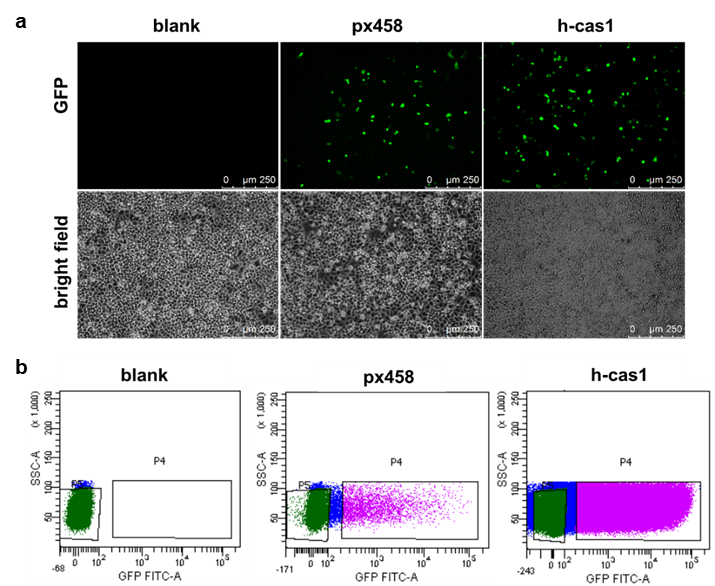
**

**Supplementary 5**


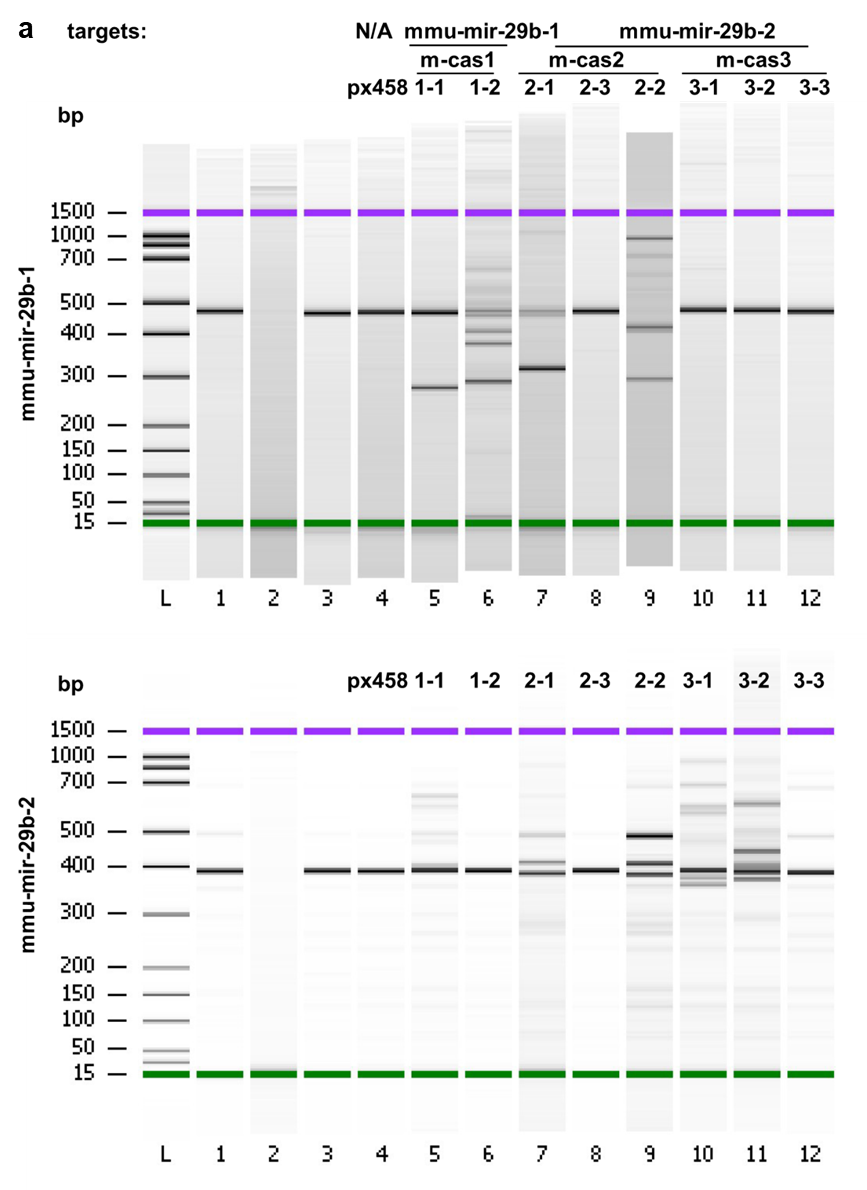


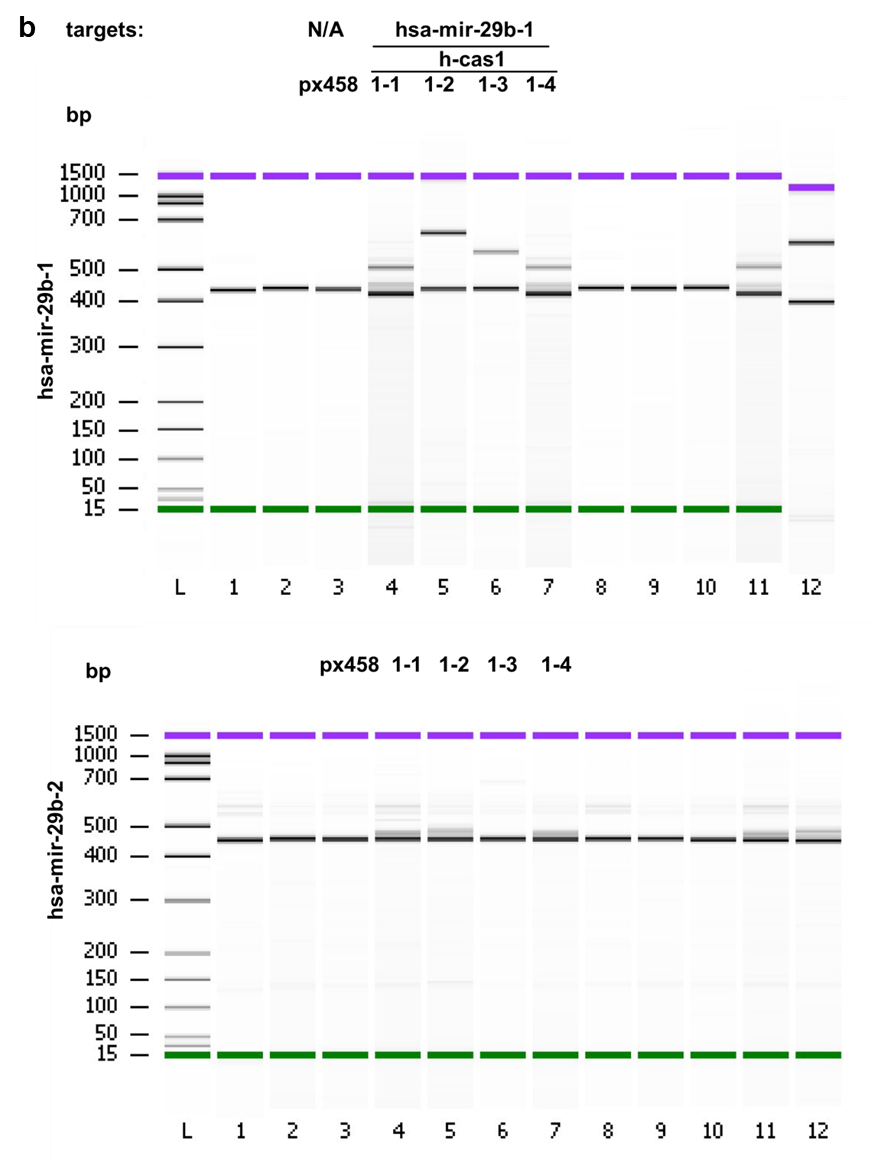


**Supplementary 6**

**
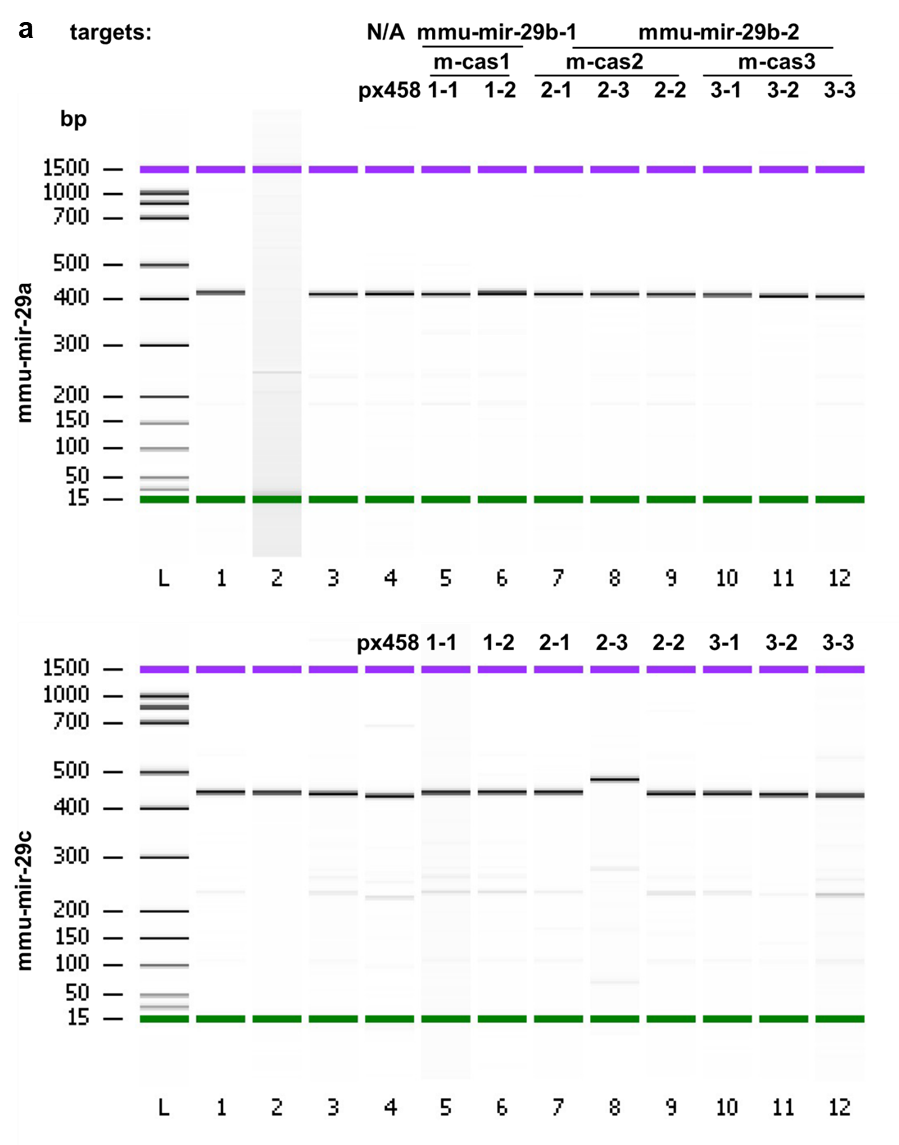
**

**
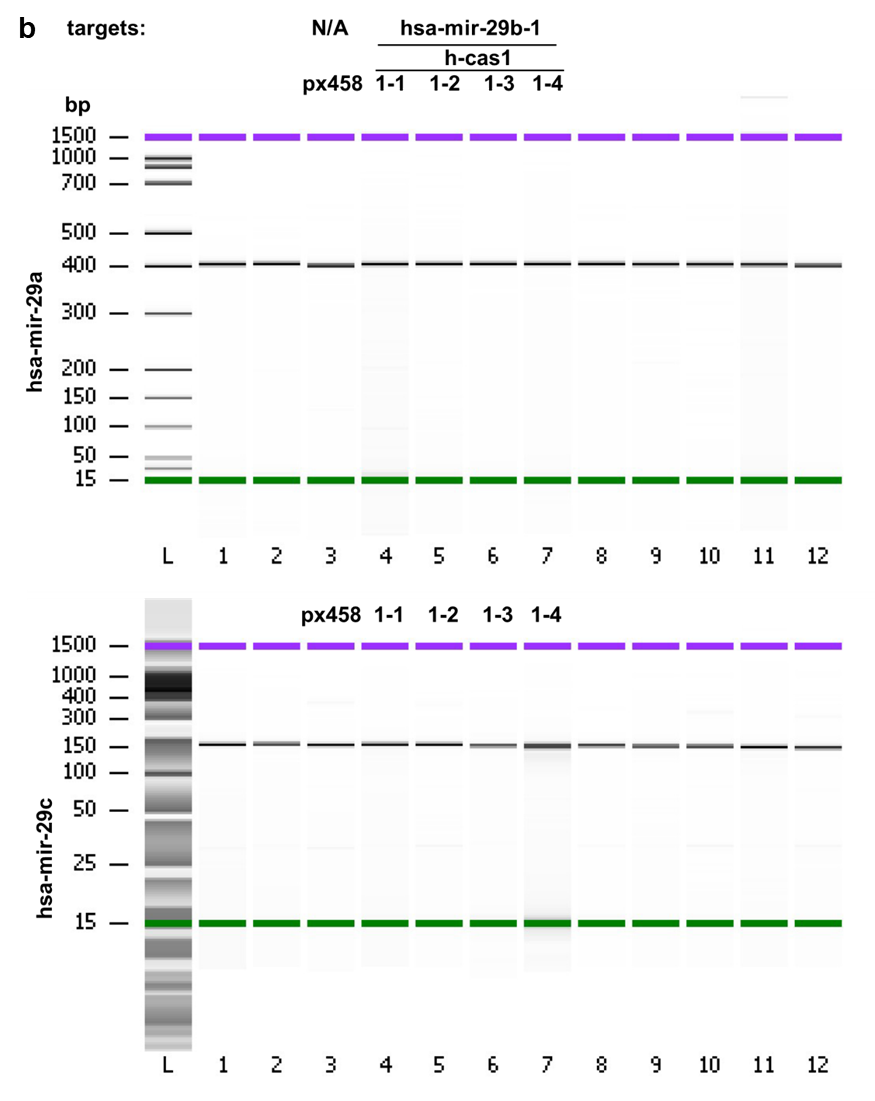
**
